# Lack of 2'-*O*-methylation in the tRNA anticodon loop of two phylogenetically distant yeast species activates the general amino acid control pathway

**DOI:** 10.1371/journal.pgen.1007288

**Published:** 2018-03-29

**Authors:** Lu Han, Michael P. Guy, Yoshiko Kon, Eric M. Phizicky

**Affiliations:** 1 Department of Biochemistry and Biophysics, Center for RNA Biology, University of Rochester School of Medicine, Rochester, NY, United States of America; 2 Department of Chemistry and Biochemistry, Northern Kentucky University, Highland Heights, KY, United States of America; Ohio State University, UNITED STATES

## Abstract

Modification defects in the tRNA anticodon loop often impair yeast growth and cause human disease. In the budding yeast *Saccharomyces cerevisiae* and the phylogenetically distant fission yeast *Schizosaccharomyces pombe*, *trm7Δ* mutants grow poorly due to lack of 2'-*O*-methylation of C_32_ and G_34_ in the tRNA^Phe^ anticodon loop, and lesions in the human *TRM7* homolog *FTSJ1* cause non-syndromic X-linked intellectual disability (NSXLID). However, it is unclear why *trm7Δ* mutants grow poorly. We show here that despite the fact that *S*. *cerevisiae trm7Δ* mutants had no detectable tRNA^Phe^ charging defect in rich media, the cells constitutively activated a robust general amino acid control (GAAC) response, acting through Gcn2, which senses uncharged tRNA. Consistent with reduced available charged tRNA^Phe^, the *trm7Δ* growth defect was suppressed by spontaneous mutations in phenylalanyl-tRNA synthetase (PheRS) or in the pol III negative regulator *MAF1*, and by overexpression of tRNA^Phe^, PheRS, or EF-1A; all of these also reduced GAAC activation. Genetic analysis also demonstrated that the *trm7Δ* growth defect was due to the constitutive robust GAAC activation as well as to the reduced available charged tRNA^Phe^. Robust GAAC activation was not observed with several other anticodon loop modification mutants. Analysis of *S*. *pombe trm7* mutants led to similar observations. *S*. *pombe* Trm7 depletion also resulted in no observable tRNA^Phe^ charging defect and a robust GAAC response, and suppressors mapped to PheRS and reduced GAAC activation. We speculate that GAAC activation is widely conserved in *trm7* mutants in eukaryotes, including metazoans, and might play a role in *FTSJ1*-mediated NSXLID.

## Author summary

The ubiquitous tRNA anticodon loop modifications have important but poorly understood functions in decoding mRNAs in the ribosome to ensure accurate and efficient protein synthesis, and their lack often impairs yeast growth and causes human disease. Here we investigate why ribose methylation of residues 32 and 34 in the anticodon loop is important. Mutations in the corresponding methyltransferase Trm7/FTSJ1 cause poor growth in the budding yeast *Saccharomyces cerevisiae* and near lethality in the evolutionarily distant fission yeast *Schizosaccharomyces pombe*, each due to reduced functional tRNA^Phe^. We previously showed that tRNA^Phe^ anticodon loop modification in yeast and humans required two evolutionarily conserved Trm7 interacting proteins for Cm_32_ and Gm_34_ modification, which then stimulated G_37_ modification. We show here that both *S*. *cerevisiae* and *S*. *pombe trm7Δ* mutants have apparently normal tRNA^Phe^ charging, but constitutively activate a robust general amino acid control (GAAC) response, acting through Gcn2, which senses uncharged tRNA. We also show that *S*. *cerevisiae trm7Δ* mutants grow poorly due in part to constitutive GAAC activation as well as to the uncharged tRNA^Phe^. We propose that *TRM7* is important to prevent constitutive GAAC activation throughout eukaryotes, including metazoans, which may explain non-syndromic X-linked intellectual disability associated with human *FTSJ1* mutations.

## Introduction

During biogenesis, tRNAs acquire extensive post-transcriptional modifications that are important for their function as an adaptor molecule during translation. Modifications in the main body of the tRNA generally affect folding or stability of specific tRNAs [1–3], whereas modifications in and around the anticodon loop play crucial roles in translation, including promoting accuracy in charging [4, 5], reading frame maintenance [6–9] and decoding [10–13]. Indeed, modification is particularly extensive in the anticodon loop region comprising the loop itself and the 31–39 closing base pair, with an average of 2.72 modifications per eukaryotic cytoplasmic tRNA [14].

Defects in anticodon loop modification frequently lead to impaired growth in the yeast *Saccharomyces cerevisiae* and to a number of human disorders, particularly neurological disorders or mitochondrial syndromes [15, 16]. For example, yeast *TAD2* and *TAD3* are required for inosine modification of the wobble nucleotide A_34_ and are essential [10], and a mutation in the corresponding human *ADAT3* gene is associated with intellectual disability and strabismus [17]. Similarly, yeast *pus3Δ* mutants have growth defects due to lack of pseudouridine (Ψ) at U_38_ and U_39_ and are temperature sensitive due to tRNA^Gln(UUG)^ [18], and a mutation in the corresponding human *PUS3* gene is associated with syndromic intellectual disability and reduced pseudouridine [19]. In addition, yeast elongator mutants lacking the carbonylmethyl-U_34_ family of modifications (xcm^5^U_34_) have a number of phenotypes due to reduced function of two or three tRNA species [20–22], while *Caenorhabditis elegans* elongator mutants are associated with neurological and developmental dysfunctions [23], and human elongator mutations are linked to familial dysautonomia [24–26]. Although the molecular mechanisms linking tRNA modification defects to human diseases remain largely unknown, the causes are amenable to study in model organisms.

One such unsolved problem is why it is important for eukaryotes to have 2'-*O*-methylated C_32_ (Cm) and N_34_ (Nm) in their tRNAs, catalyzed by Trm7 family members. In *S*. *cerevisiae*, a *trm7Δ* mutant grows poorly due to reduced function of tRNA^Phe^, but not its other two substrates, tRNA^Leu(UAA)^ and tRNA^Trp(CCA)^, and in the phylogenetically distant yeast *Schizosaccharomyces pombe*, the near lethal phenotype of a *trm7Δ* mutant is rescued by overproduction of tRNA^Phe^ [27–29]. In humans, seven different alleles of the human *TRM7* homolog *FTSJ1* have been linked to non-syndromic X-linked intellectual disability (NSXLID) [30–34], and lymphoblastoid cell lines (LCLs) derived from patients with two different *FTSJ1* alleles had tRNA^Phe^ with undetectable levels of Cm_32_ and Gm_34_ [34].

In eukaryotes, modification of tRNAs by Trm7 involves conserved partner proteins for each modification and a conserved circuitry for tRNA^Phe^ anticodon loop modification. In *S*. *cerevisiae*, Trm7 interacts separately with Trm732 and Trm734 for formation of Cm_32_ and Nm_34_ respectively in each of its three tRNA substrates, and the presence of Cm_32_ and Gm_34_ in tRNA^Phe^ drives the formation of wybutosine (yW) from 1-methylguanosine (m^1^G) modification at G_37_ [28]. *S*. *pombe* Trm732 and Trm734 have the same functions in Cm_32_ and Gm_34_ modification of tRNA^Phe^ and, as in *S*. *cerevisiae*, Cm_32_ and Gm_34_ drive formation of yW_37_ in tRNA^Phe^ [29]. Moreover, available evidence suggests that this circuitry is conserved in humans. tRNA^Phe^ from patient LCLs with an *FTSJ1* deletion or a splice site mutation had substantially reduced peroxywybutosine (o2yW_37_), as expected if o2yW_37_ formation is stimulated by Cm_32_ and Gm_34_ [34]. Furthermore, expression of either *FTSJ1* or the *TRM732* ortholog *THADA* complements the corresponding *S*. *cerevisiae* mutants, as does expression of *S*. *pombe trm7*^*+*^ and the *Drosophila TRM7* homolog ORF CG5220 [29].

However, despite the extensive studies of Trm7 in different organisms, the biological consequences of lacking Cm_32_ and Gm_34_ modifications on tRNA^Phe^ remain unclear. We investigate here why Cm_32_ and Gm_34_ modifications are critical for tRNA^Phe^ function and healthy growth in yeast. We provide evidence that despite the lack of an obvious charging defect, *trm7Δ* mutants activate a robust general amino acid control (GAAC) response in both *S*. *cerevisiae* and *S*. *pombe*, each in a manner suggesting the sensing of uncharged tRNA. Moreover, in each organism we find that suppressors of the *trm7Δ* growth defect frequently map to subunits of phenylalanyl tRNA synthetase (PheRS) and reduce the GAAC response toward that in wild type cells. These results argue for a conserved Trm7 biology in eukaryotes and argue that subtle changes in tRNA^Phe^ charging have dramatic effects on cell physiology.

## Results

### Suppressors of the growth defect of *S*. *cerevisiae trm7Δ* mutants map to PheRS, despite the lack of an obvious charging defect

To begin to elucidate why Trm7 and 2’-*O*-methylation at C_32_ and N_34_ of tRNAs were important, we isolated and analyzed spontaneous suppressors that improved the slow growth phenotype of *S*. *cerevisiae trm7Δ* mutants. This slow growth phenotype is apparent by analysis of growth of *trm7Δ* [*URA3 CEN TRM7*] mutants on media containing 5-FOA [28], and by growth analysis of *trm7Δ* mutants on rich media and minimal media immediately after loss of the [*URA3 CEN TRM7*] plasmid (S1 Fig), and in all of these conditions, the growth defect is fully suppressed by overproduction of tRNA^Phe^ (S1 Fig, [28]). We isolated 21 genetically independent faster growing suppressors after plating *trm7Δ* cells on YPD (rich) medium, and found that 19 of them had a dominant mutation in either *FRS1* or *FRS2* (S1 Table), which encode the two subunits of PheRS [35].

**Fig 1:**
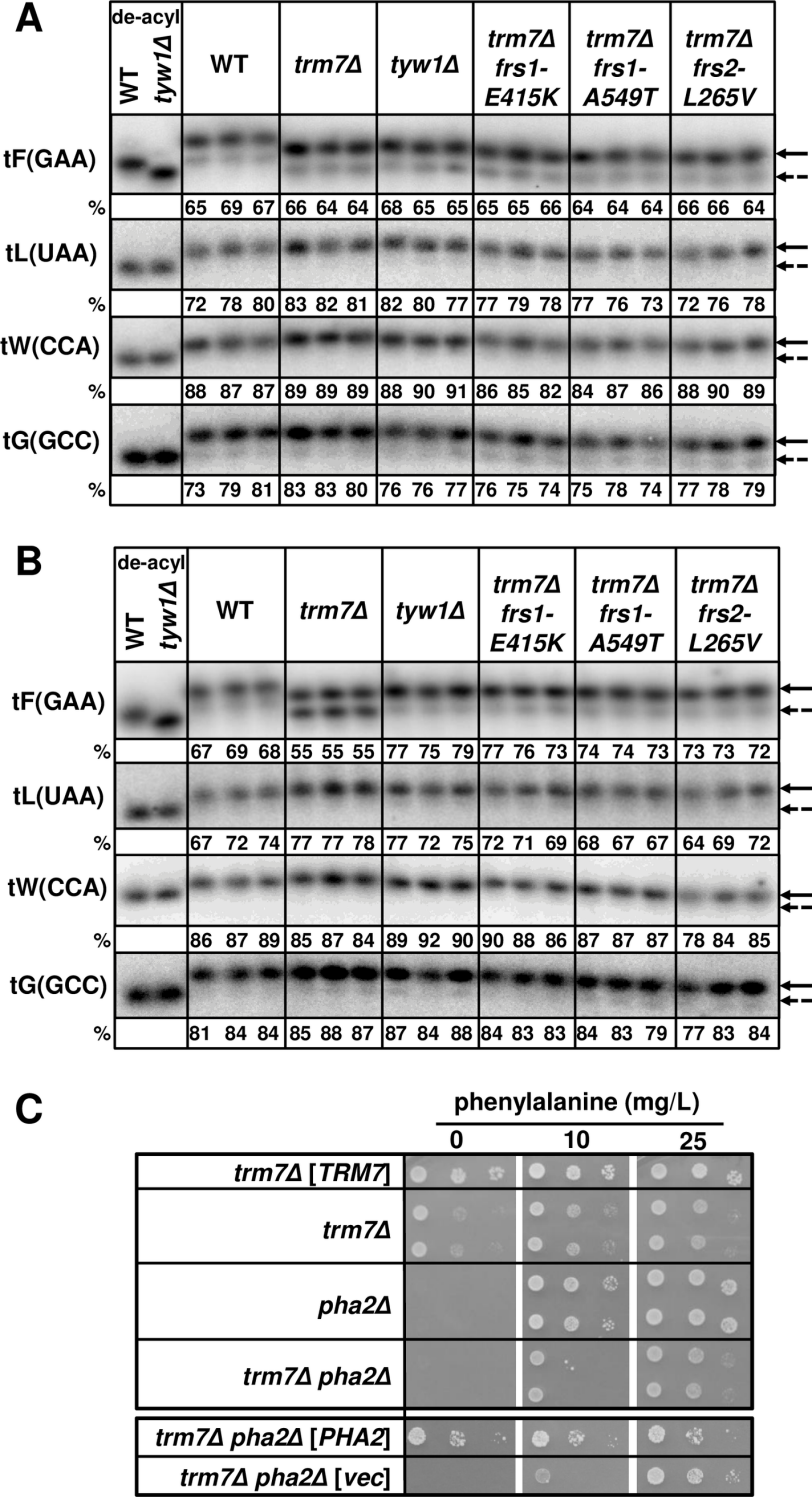
tRNAs from *trm7Δ* mutants have no obvious charging defect when grown in rich media, and a prominent charging defect when grown in synthetic minimal media. Strains as indicated were grown in rich media or minimal media, and RNA was isolated under acidic conditions to maintain tRNA charging, and analyzed by Northern blot as described in Materials and Methods, with hybridization probes as indicated. Control samples (WT and *tyw1Δ*) were treated with mild base to de-acylate the tRNA. Upper arrows denote charged tRNA species, and lower arrows with dashed lines denote uncharged tRNA species. Numbers below each sample indicate percentage of charged tRNA. ***(A)* Analysis of *trm7Δ* mutants grown in rich media. (*B*) Analysis of *trm7Δ* mutants grown in minimal media. (*C*) Limiting phenylalanine exacerbates the *trm7Δ* growth defect in minimal media.** Strains as indicated were grown overnight in minimal complete media at 30°C, washed with water once, diluted to OD_600_ of ∼0.5, serially diluted 10-fold in water, and 2 μL was spotted onto SD-Phe media containing different concentrations of phenylalanine as indicated, and incubated at 30°C for 4 to 5 days.

This result was surprising since we had shown previously that tRNA^Phe^ from *trm7Δ* mutants had no obvious charging defects, and was present at similar overall levels in WT cells [28]. Indeed, analysis of tRNA isolated under acidic conditions to preserve charging [36, 37] showed that tRNA^Phe^ from three independent freshly derived *trm7Δ* isolates grown in rich media had no discernible charging defect (65 ± 1% charging), compared to tRNA^Phe^ from WT cells (67 ± 2%) or *tyw1Δ* mutants (66 ± 2%) (Fig 1A). *tyw1Δ* mutants, like *trm7Δ* mutants, have m^1^G_37_ instead of yW_37_ [38], and migrate identically on acidic gels. Similarly, no charging defect was seen in the other two Trm7 substrates, tRNA^Leu(UAA)^ and tRNA^Trp^, or in the non-substrate tRNA^Gly(GCC)^. Furthermore, no increase in charging was observed in each of three representative suppressors of the *trm7Δ* growth defect (*frs1-E415K*, *frs1-A549T*, and *frs2-L265V*) for any of the tRNA species examined (65 ± 1%, 64 ± 0%, 65 ± 1% respectively for tRNA^Phe^). By contrast, in synthetic minimal medium a more prominent charging defect was observed by acidic Northern analysis of tRNA^Phe^ from *trm7Δ* cells (Fig 1B). Under this growth condition, we found that tRNA^Phe^ charging levels were reduced to 55 ± 0% in *trm7Δ* mutants, substantially below those of WT cells (68 ± 1%) and *tyw1Δ* mutants (77 ± 2%). Moreover, the three *trm7Δ* suppressors all restored tRNA^Phe^ charging to levels similar to charging observed in *tyw1Δ* mutants (75 ± 2%, 74 ± 1%, 73 ± 1% respectively for the *frs1-E415K*, *frs1-A549T*, and *frs2-L265V* mutants).

Consistent with a tRNA^Phe^ charging defect in minimal media, we found that limiting phenylalanine exacerbated *trm7Δ* growth defects. After deletion of *PHA2* (encoding prephenate dehydratase) to confer phenylalanine auxotrophy [39], we found that *trm7Δ pha2Δ* mutants showed an exacerbated growth defect compared to *trm7Δ* mutants in the presence of 10 mg/L phenylalanine, and this defect was complemented by re-introduction of the *PHA2* gene on a plasmid; by contrast, under the same conditions *pha2Δ* mutants showed no discernable growth defect compared to a WT (*trm7Δ* [*TRM7*]) strain (Fig 1C).

### *S*. *cerevisiae trm7Δ* mutants activate a robust general amino acid control response through Gcn2

**Fig 2:**
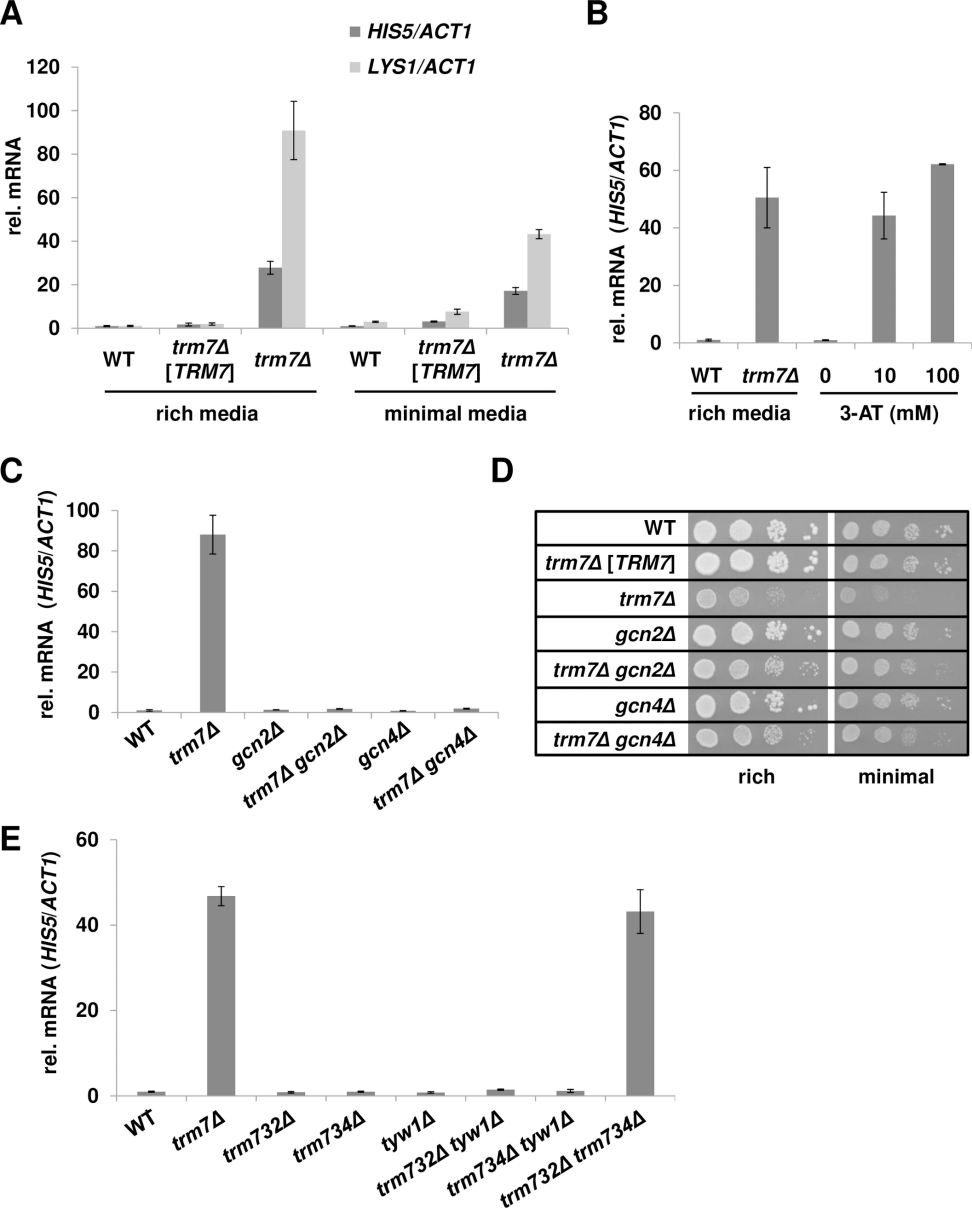
*trm7Δ* mutants grown in either rich media or synthetic minimal media activate a robust general amino acid control (GAAC) response, mediated by Gcn2. **(*A*) *trm7Δ* mutants grown in rich media and minimal media activate the GAAC response.** Strains as indicated were grown in either rich media or minimal media at 30°C to mid-log phase, and mRNA was isolated and analyzed by RT-qPCR. The mRNA levels of *GCN4*-regulated genes, *LYS1* and *HIS5*, were normalized to those of the nonregulated *ACT1*, and then normalized to WT grown in rich media. **(*B*) Activation of the GAAC response in *trm7Δ* mutants is comparable to that of WT strains treated with 3-AT.** WT and *trm7Δ* strains were grown in rich media to mid-log phase. For 3-AT treatment, the WT His^+^ strains were grown in synthetic complete media to mid-log phase, spun down, resuspended in SD-His media containing different concentrations of 3-AT for 1h, and then mRNA was isolated and *HIS5* mRNA was analyzed by RT-qPCR, as in (*A*). **(*C*) Activation of the GAAC response in *trm7Δ* mutants is Gcn2 dependent.** Strains as indicated were grown in rich media and analyzed for relative *HIS5* mRNA levels as in (*A*). **(*D*) Deletion of *GCN2* or *GCN4* mildly suppresses *trm7Δ* growth defects.** Strains as indicated were grown overnight in rich medium, and analyzed by serial dilution, spotting to rich (YPD) or minimal media (SD complete), and incubation for 4 d at 25°C. **(*E*) *trm732Δ trm734Δ* mutants phenocopy *trm7Δ* mutants to induce the GAAC response.** Strains with deletions of *TRM732*, *TRM734*, or *TYW1*, or with combinations of deletions, were grown in rich media at 30°C to mid-log phase, and mRNA was isolated and analyzed for relative *HIS5* levels as in (*A*).

Since acidic Northern analysis of *trm7Δ* mutants revealed no detectable tRNA^Phe^ charging defect in rich media, but a distinct charging defect in minimal media that was suppressed by each of three suppressors, we examined *in vivo* charging in both rich and minimal media by analysis of the general amino acid control (GAAC) response [40]. In yeast and other eukaryotes, uncharged tRNAs arising from amino acid starvation or lack of functional tRNA synthetases bind to Gcn2 and activate its kinase domain, resulting in phosphorylation of eIF2α, de-repression of *GCN4* translation, and transcriptional activation of nearly one tenth of the yeast genome, including numerous amino acid biosynthetic genes [41–43]. We reasoned that if there was a subtle accumulation of uncharged tRNA^Phe^ in *trm7Δ* mutants, this might result in a GAAC response. Indeed, RT-qPCR analysis of mRNA from cell pellets collected from the same cultures as those used in the acidic Northerns (Fig 1A and 1B) revealed that the mRNA levels of two known *GCN4* target genes, *HIS5* and *LYS1*, were significantly increased in *trm7Δ* mutants (relative to *ACT1*), compared to WT cells. In rich media, relative levels of *HIS5* and *LYS1* mRNA increased 27.8-fold and 90.9-fold respectively, and in minimal media relative levels increased 17.1-fold and 43.2-fold (Fig 2A, S2 Table). These GAAC activation levels in *trm7Δ* mutants were comparable to those in WT His^+^ cells treated for 1 hour with 10 mM or 100 mM 3-amino-1,2,4-triazole (3-AT) (Fig 2B, S2 Table), a competitive inhibitor of His3 that has been used extensively to induce the yeast GAAC response [40, 44]. This robust constitutive GAAC response in *trm7Δ* mutants provided initial evidence that charged tRNA was limiting in vivo.

Further analysis showed that the *trm7Δ*-mediated induction of the GAAC response is occurring through Gcn2, which senses uncharged tRNA [45]. The GAAC pathway can be induced by Gcn2 or by a pathway independent of Gcn2 [46–48], which is not well understood. As expected of the Gcn2-mediated GAAC response, we found that *trm7Δ gcn2Δ* strains completely abolished transcriptional activation of the *HIS5* gene, as did the control *trm7Δ gcn4Δ* mutants (Fig 2C, S2 Table). These results provided compelling evidence that the GAAC response observed in *trm7Δ* mutants arose from uncharged tRNA. We note that the slow growth of *S*. *cerevisiae trm7Δ* mutants appears to be due to both lack of available charged tRNA^Phe^ and to activation of the GAAC response itself, since either a *gcn2Δ* or a *gcn4Δ* mutation partially improved growth of a *trm7Δ* strain in both rich and minimal media (Fig 2D). Nonetheless, the increased stress on *trm7Δ* mutants associated with activation of the GAAC response must be a secondary consequence of the lack of available charged tRNA^Phe^ required to initiate the response.

Further analysis demonstrated that activation of the GAAC response was closely tied to the growth phenotype of *trm7Δ* related strains. Thus, as measured by *HIS5* mRNA levels, the GAAC pathway was not activated by the lack of Cm_32_ in a *trm732Δ* mutant, by lack of Nm_34_ in a *trm734Δ* mutant, or by lack of yW_37_ in a *tyw1Δ* mutant, or by a *trm732Δ tyw1Δ* double mutant or a *trm734Δ tyw1Δ* double mutant (Fig 2E, S2 Table), all of which are healthy strains [28]. By contrast, a *trm732Δ trm734Δ* strain fully activated the GAAC response, with relative *HIS5* mRNA levels comparable to those of *trm7Δ* mutants, consistent with our previous finding that *trm732Δ trm734Δ* strains phenocopied the growth defect of *trm7Δ* mutants [28].

### Suppressors of the *S*. *cerevisiae trm7Δ* growth defect reduce activation of the GAAC response

**Fig 3:**
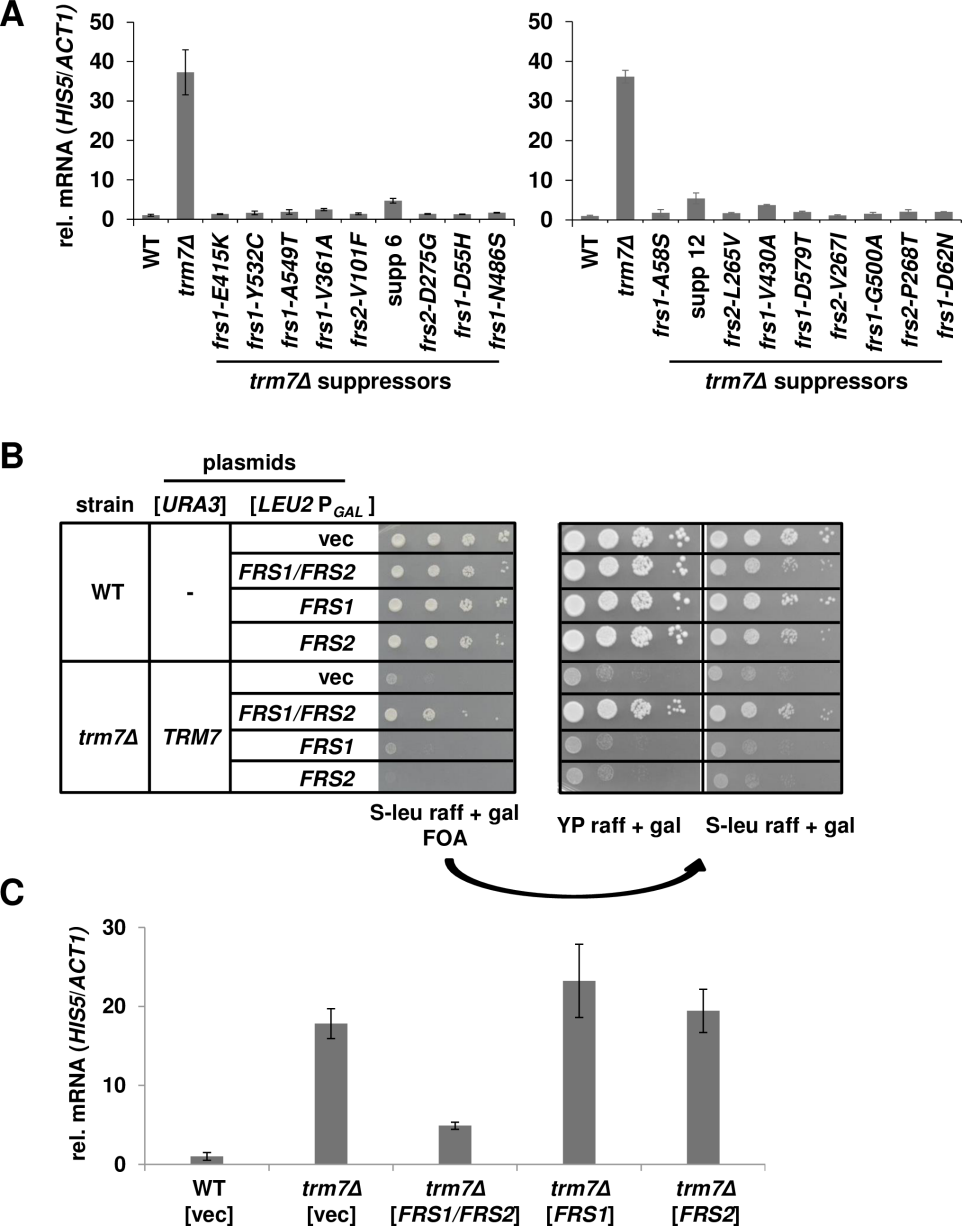
Most suppressors of the *S*. *cerevisiae trm7Δ* growth defect map to PheRS subunits and all reduce activation of the GAAC response. **(*A*) Each of 18 suppressors of the *trm7Δ* growth defect examined has reduced GAAC induction.** Strains were grown in rich media at 30°C to mid-log phase, and mRNA was isolated and analyzed for relative *HIS5* mRNA levels. **(*B*) Overproduction of both subunits of PheRS partially suppresses the *trm7Δ* growth defect.** WT or *trm7Δ* [*URA3 TRM7*] strains containing a high copy [2μ *LEU2*] plasmid expressing *FRS1*, *FRS2*, both, or neither as indicated under P_*GAL*_ control were grown overnight in S-Leu medium containing raffinose and analyzed by spotting to synthetic media containing 5-FOA, raffinose (raff), and galactose (gal), and incubated for 3 d at 30°C. Strains from the 5-FOA plate were then purified on the same medium, grown overnight in S-Leu medium containing raffinose and galactose, diluted, and spotted to rich (YP) media and minimal (S- leu) as indicated, and incubated for 3 d at 30°C. **(*C*) Overproduction of both subunits of PheRS reduces the GAAC induction in *trm7Δ* cells.** Strains containing a [*LEU2*] plasmid expressing *FRS1*, *FRS2*, both, or neither as indicated under P_*GAL*_ control were grown in S-Leu medium containing raffinose and galactose, and then mRNA was isolated and relative *HIS5* mRNA levels were determined.

Strikingly, each of 18 *trm7Δ* suppressors we examined reduced the magnitude of the GAAC response from relative *HIS5* mRNA levels of 37.3-fold and 36.1-fold in *trm7Δ* mutants (Fig 3A, left side and right side respectively, S3 Table) to levels approaching those observed in WT cells (1.1- to 5.5-fold). These suppressors included 16 with mutations in PheRS subunits, as well as two that did not have mutations in PheRS. The co-reversion of the *trm7Δ* growth defect and the GAAC response further implied that lack of available charged tRNA was the cause of the growth defect.

Consistent with the interpretation that the poor growth of *trm7Δ* mutants is caused by defective charging, we found that overproduction of PheRS on a [P_*GAL*_-*FRS1* P_*GAL*_-*FRS2*] plasmid improved *trm7Δ* growth, compared to that of a *trm7Δ* strain with a plasmid expressing either subunit of PheRS, or an empty vector (Fig 3B). Furthermore, overexpression of both *FRS1* and *FRS2* improved tRNA^Phe^ charging (S2 Fig) and reduced relative *HIS5* mRNA levels in *trm7Δ* mutants from 17.8 to 4.9, while overexpression of either *FRS1* or *FRS2* had no effect (Fig 3C, S3 Table).

### Other genetic manipulations expected to modulate availability of charged tRNA^Phe^ levels also suppress the growth defect and reduce GAAC activation

**Fig 4:**
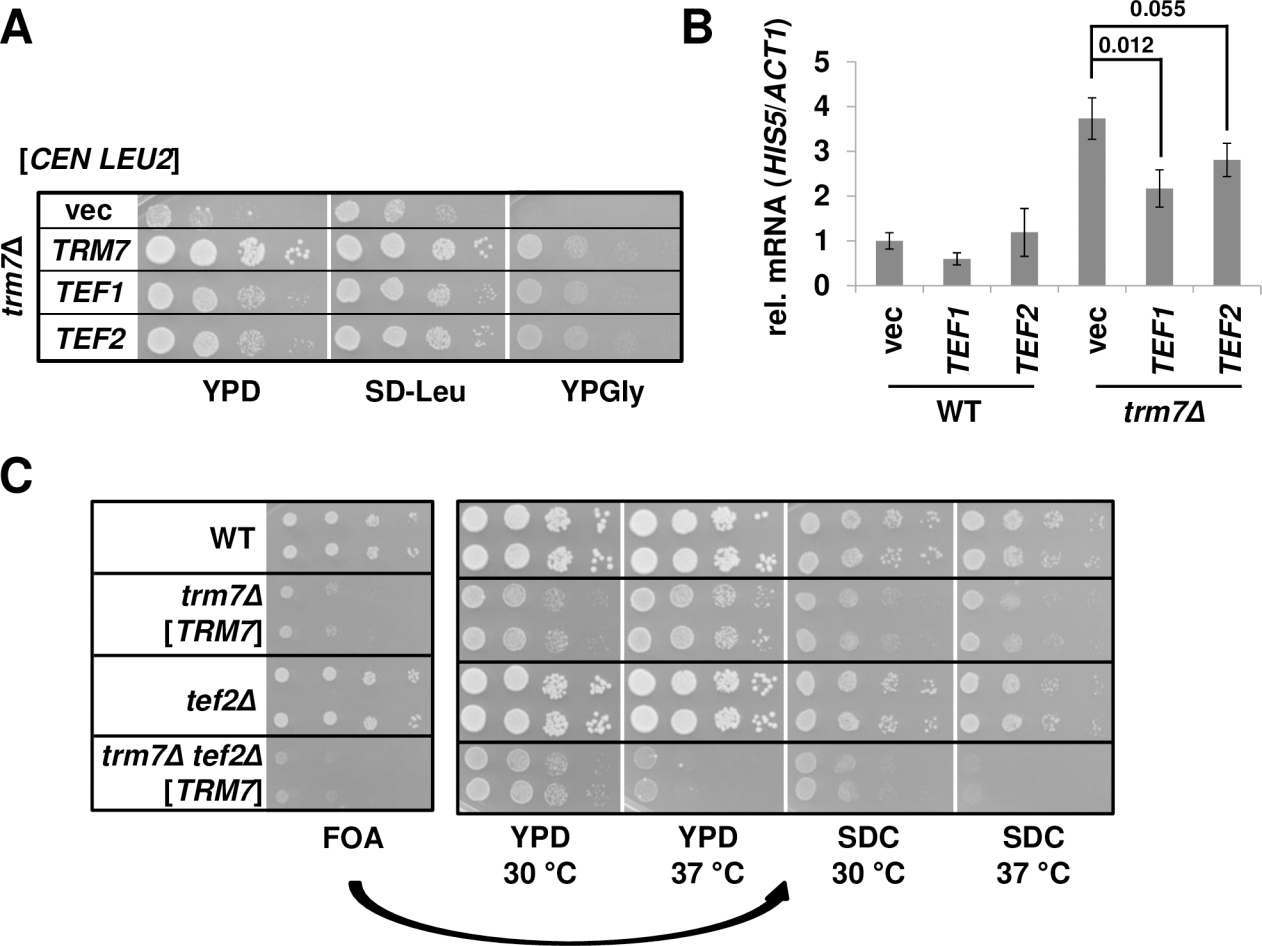
EF-1A levels commensurately affect both *trm7Δ* growth and activation of the GAAC response. **(*A*) An additional copy of EF-1A partially suppress the growth defect of *trm7Δ* mutants.** *trm7Δ* mutants containing a [*CEN LEU2*] plasmid with *TEF1*, *TEF2*, or *TRM7* as indicated were grown overnight in SD-Leu at 30°C, analyzed by spotting to plates as indicated, and incubated for 2 d at 30°C. **(*B*) An additional copy of EF-1A partially suppresses induction of the GAAC response.** WT or *trm7Δ* strains containing a [*CEN LEU2*] plasmid with *TEF1* or *TEF2* as indicated were grown as in (*A*), and relative *HIS5* mRNA levels were determined. p values are shown above. **(*C*) Reduced levels of EF-1A amplify the growth defect of *trm7Δ* mutants.** WT and *trm7Δ* [*CEN URA3 TRM7*] strains with or without a *tef2Δ* mutation, as indicated, were grown overnight in rich media, and analyzed by spotting to media containing 5-FOA, and incubation for 2 d at 30°C. Strains from the 5-FOA plate were then purified on medium containing 5-FOA, grown overnight in YPD, diluted, spotted on rich (YPD) and minimal (SD complete) plates as indicated, and incubated for 2 d at indicated temperatures.

Since elongation factor 1A (EF-1A) binds to and delivers aminoacylated tRNA to the ribosomes A-site, we speculated that its overexpression might result in more charged tRNA^Phe^ available for use in translation, thereby improving *trm7Δ* growth. To test this hypothesis, we introduced an extra copy of *TEF1* or *TEF2*, which encode identical copies of EF-1A, into a *trm7Δ* strain. We found that elevated levels of EF-1A moderately rescued the growth defect (Fig 4A), and partially suppressed the GAAC activation, with p values of 0.012 and 0.055 respectively (Fig 4B, S4 Table), while deletion of *TEF2* in a *trm7Δ* strain exacerbated the slow-growth phenotype (Fig 4C). The improved growth of *trm7Δ* mutants with increased EF-1A levels presumably reflects increased availability of aminoacylated tRNA^Phe^ for translation after charging by PheRS, rather than increased tRNA^Phe^ charging, which is not significantly altered (S3 Fig).

**Fig 5:**
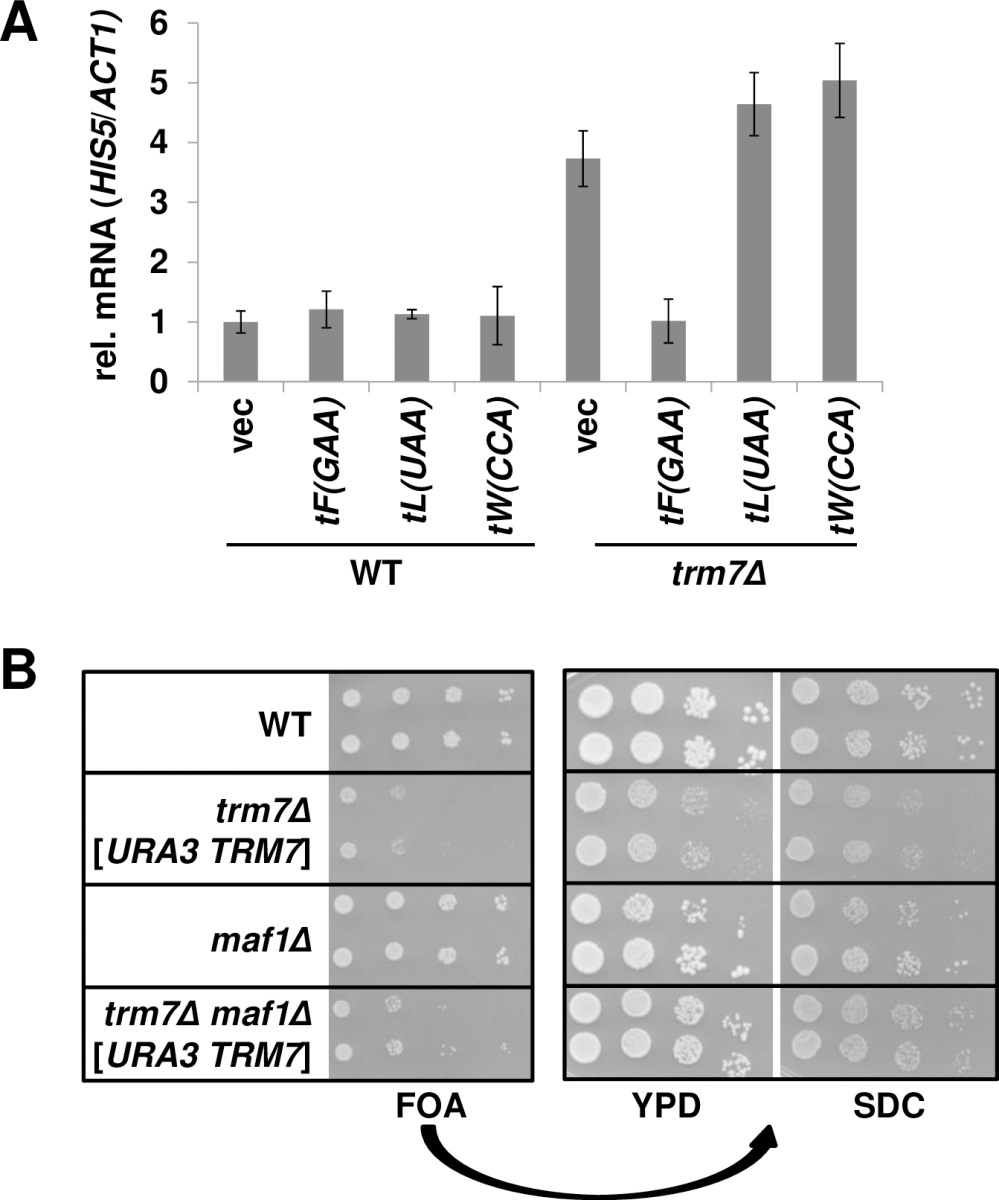
Manipulations expected to increase tRNA^Phe^ levels link suppression of the *trm7Δ* growth defect with reduction of GAAC induction. **(*A*) Overexpression of tRNA**^**Phe(GAA)**^ **reduces induction of the GAAC response.** WT or *trm7Δ* strains containing a high-copy [2μ *LEU2*] plasmid expressing *tF(GAA)*, *tL(UAA)*, *tW(CCA)* or a vector as indicated were grown in SD-Leu at 30°C to mid-log phase, and mRNA was isolated and analyzed for relative *HIS5* mRNA levels. **(*B*) A *maf1Δ* mutation suppresses the growth defect of a *trm7Δ* mutant.** WT and *trm7Δ* [*CEN URA3 TRM7*] strains with or without a *maf1Δ* mutation, as indicated, were grown overnight in rich media, and analyzed by spotting to media containing 5-FOA, and incubation for 2 d at 30°C. Strains from the 5-FOA plate were then purified on medium containing 5-FOA, grown overnight in YPD, diluted, spotted on plates as indicated, and incubated for 2 d at 30°C.

Similar results are obtained by treatments expected to increase the population of charged tRNA^Phe^. Thus, overexpression of tRNA^Phe^ on a high copy plasmid, which is known to suppress the *trm7Δ* growth defect (S1 Fig, [28]) and results in 4.2-fold overproduction of tRNA^Phe^ (S4A Fig), reduced the relative *HIS5* mRNA levels to values similar to WT cells, while overexpression of other control tRNAs had no effect (Fig 5A, S5 Table). Furthermore, whole genome sequencing showed that *trm7Δ* suppressor 12 (Fig 3A) had a mutation in *MAF1*, a negative regulator of pol III transcription [49]. Since this *maf1-C299Y* mutation alters a highly conserved residue in the Box C region of Maf1, we inferred that this mutation behaved as a null mutation [49–51]. To test this inference, we introduced a *MAF1* deletion into the *trm7Δ* [*URA3 TRM7*] strains and tested for growth on media containing 5-FOA to select against the *URA3* plasmid. The resulting *trm7Δ maf1Δ* strain grew better than the control *trm7Δ* mutants (Fig 5B), and had increased levels of tRNA^Phe^ and tRNA^Phe^ charging, in both log phase and stationary phase (S4B Fig).

### Robust activation of the GAAC response is not routinely observed in mutants defective in anticodon loop modifications

**Fig 6:**
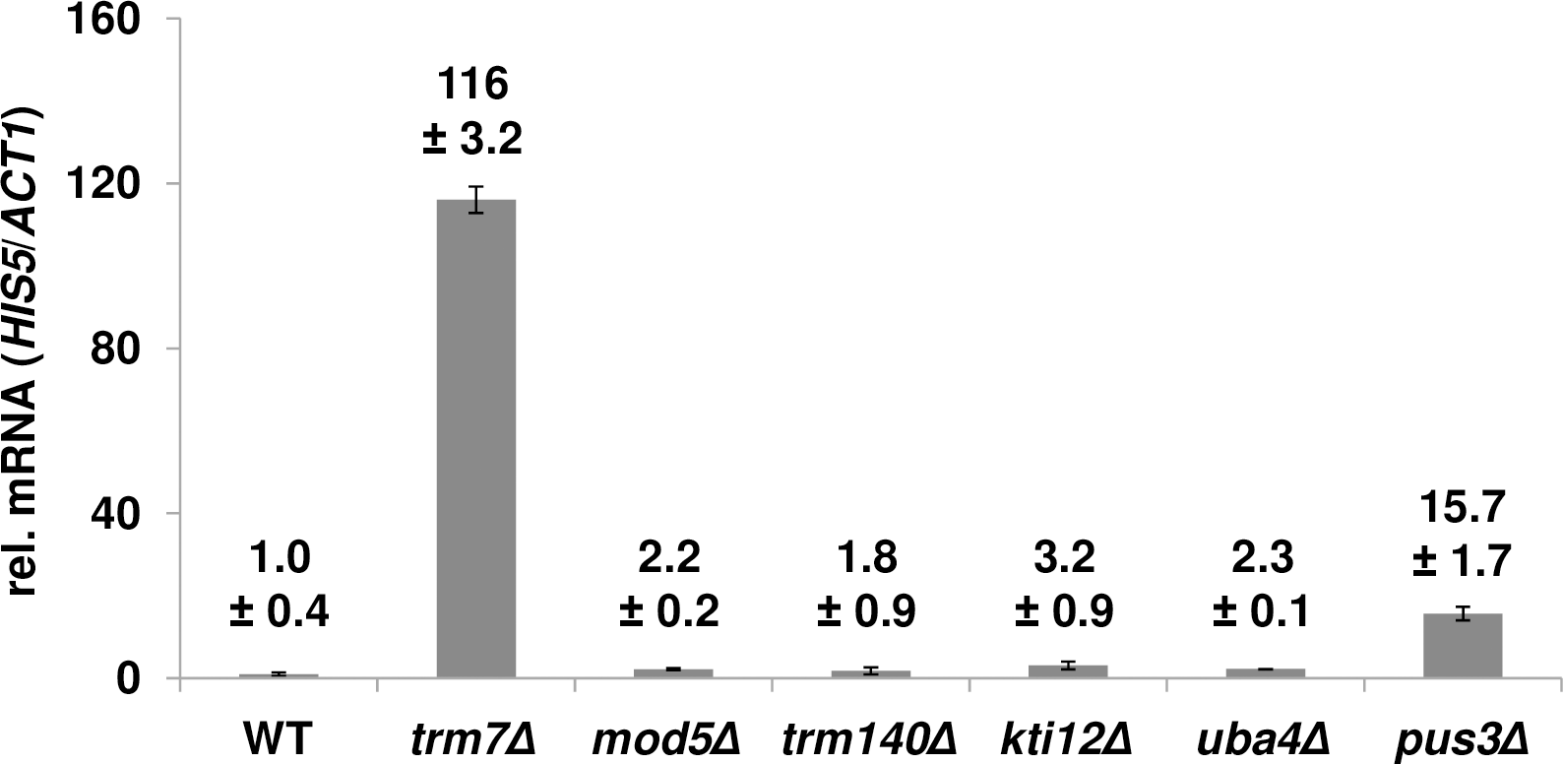
Robust GAAC activation in *trm7Δ* mutants is not a general theme among anticodon loop modification mutants. Strains with *trm7Δ*, *mod5Δ*, *trm140Δ*, *kti12Δ*, *uba4Δ*, or *pus3Δ* mutations in anticodon loop modification genes, as indicated, were grown in rich media at 30°C to mid-log phase, and then mRNA was isolated and analyzed for relative *HIS5* mRNA levels.

To determine if GAAC activation is a common theme among tRNA anticodon loop modification mutants, we examined the GAAC response in several other mutants, including strains lacking isopentenyladenosine (i^6^A_37_), due to a *mod5Δ* mutation [52]; 3-methylcytidine (m^3^C_32_), due to a *trm140Δ* mutation [53, 54]; the cm^5^U moiety of xcm^5^U_34_, due to a *kti12Δ* mutation [55]; the 2-thiouridine moiety (s^2^U) of mcm^5^s^2^U_34_, due to a *uba4Δ* mutation [56]; and Ψ_38_ and Ψ_39_, due to a *pus3Δ* mutation [57]. Among these mutants, only *pus3Δ* mutants had a substantial increase in relative *HIS5* mRNA levels (15.7-fold), albeit much less than in *trm7Δ* mutants (116-fold in this experiment), whereas other modification mutants had only slightly increased *HIS5* mRNA levels (1.8- to 3.2-fold) (Fig 6). Thus, robust GAAC activation, as observed in *trm7Δ* mutants, is not a general theme among modification mutants.

### *S*. *pombe* Trm7 depletion and suppressors of the *trm7Δ* growth defect parallel charging and GAAC effects in *S*. *cerevisiae*

**Fig 7:**
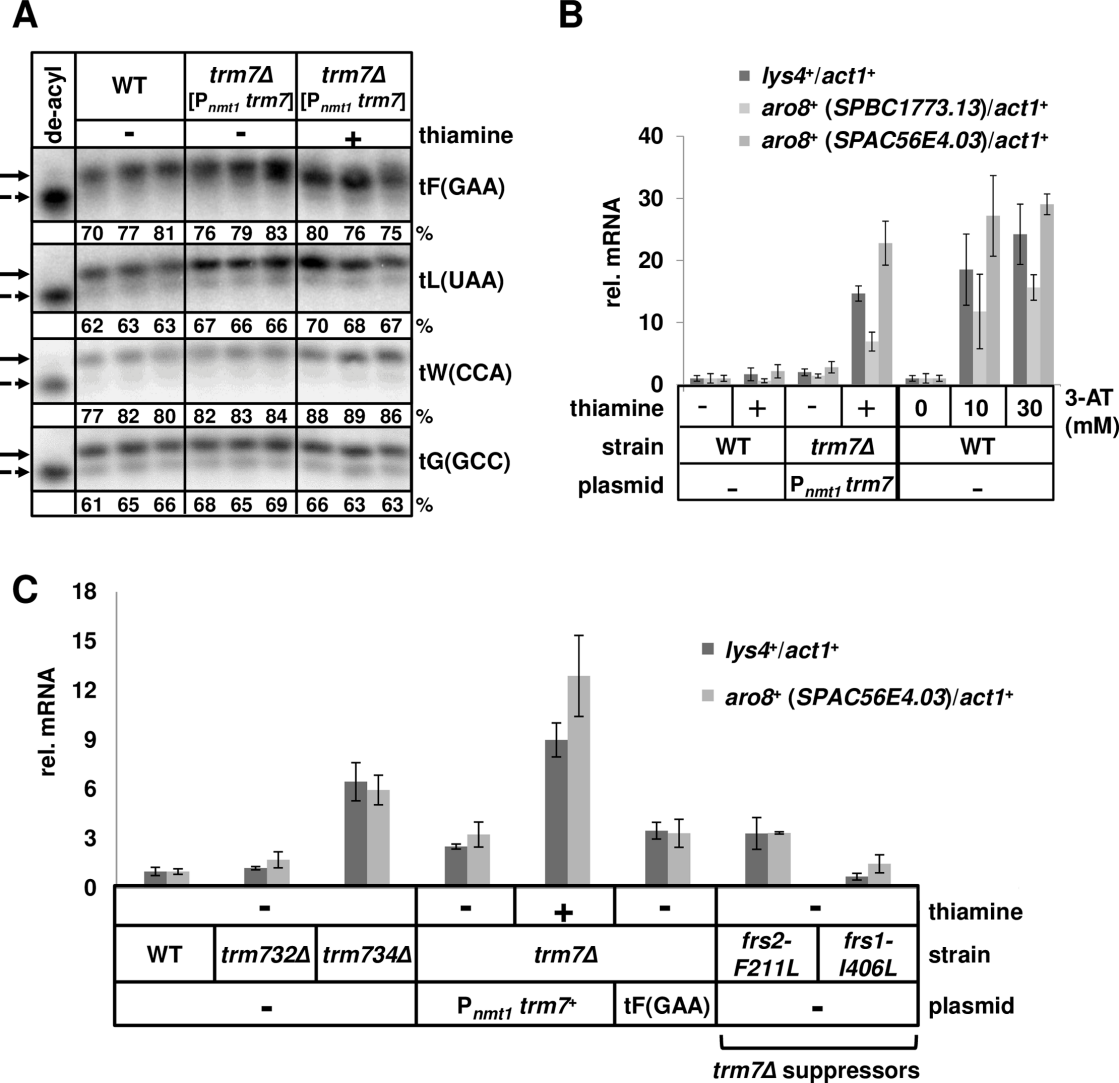
*S*. *pombe* strains depleted of Trm7 have no detectable tRNA^Phe^ charging defect but induce a robust GAAC response, which is reduced in *trm7Δ* suppressors. **(*A*) *S*. *pombe trm7Δ* [P**_***nmt1***_ ***trm7***^***+***^**] strains grown under repressive conditions have no obvious tRNA**^**Phe**^ **charging defect.** *S*. *pombe trm7Δ* [P_*nmt1*_ *trm7*^*+*^] strains were grown in EMM with thiamine (repressive conditions) or without thiamine, along with WT *S*. *pombe* grown in EMM, and then RNA was isolated under acidic conditions and analyzed for charging as in Fig 1(*A*). **(*B*) *S*. *pombe trm7Δ* [P**_***nmt1***_ ***trm7***^***+***^**] strains grown under repressive conditions induce the GAAC response.** Left side: *S*. *pombe* WT and *trm7Δ* [P_*nmt1*_ *trm7*^*+*^] strains were grown in EMM with thiamine (repressive conditions) or without thiamine to log phase and then mRNA was isolated and analyzed by RT-qPCR for mRNA levels of GAAC-regulated genes, *lys4*^*+*^, *aro8*^*+*^ (*SPBC1773*.*13*), and *aor8*^*+*^ (*SPAC56E4*.*03*), normalized to those of nonregulated *act1*^*+*^, and then normalized to WT without thiamine. Right side: GAAC induction of WT cells treated with different concentrations of 3-AT as indicated for 4 hours, and evaluated in parallel to the Left side. **(*C*) *S*. *pombe trm734Δ* mutants partially activate the GAAC response, and *trm7Δ* suppressors have mutations in PheRS and significantly reduced GAAC induction.** *S*. *pombe* strains as indicated were grown in EMM as in (*A*). mRNA was isolated from each strain and analyzed by RT-qPCR as in (*B*) for relative mRNA levels.

To investigate the evolutionary implications of our results, we examined the charging status and GAAC response in *S*. *pombe trm7Δ* mutants, which as in *S*. *cerevisiae*, grow poorly due to lack of sufficient tRNA^Phe^ [29]. Since *Sp trm7Δ* strains are barely viable, we assayed tRNA charging and GAAC induction after growth of an *Sp trm7Δ* [P_*nmt1*_ *trm7*^+^] strain in minimal (EMM) medium, followed by addition of thiamine to repress *Sp* Trm7 expression [29]. As in *S*. *cerevisiae trm7Δ* mutants grown in rich medium, we found that *Sp* tRNA^Phe^ charging levels were comparable in *Sp trm7Δ* [P_*nmt1*_ *trm7*^+^] grown in repressing conditions to deplete Trm7 (77.0 ± 2.6%), compared to the same strain in permissive conditions (79.3 ± 3.5%) or to WT strains (76 ± 5.6%) (Fig 7A). (Note that a similar *S*. *pombe* Trm7 depletion experiment could not be done in rich (YES) medium due to the presence of thiamine in this medium.) However, examination of mRNA from cell pellets collected in parallel from the same cultures revealed that *Sp trm7Δ* [P_*nmt1*_ *trm7*^+^] strains grown in repressing conditions induced the GAAC response, with significantly increased relative mRNA levels of three Gcn2 dependent GAAC-regulated genes (*lys4+*, *aro8+* (SPBC1773.13), and *aro8+* (SPAC56E4.03)) [58], compared to WT cells (14.7-fold, 6.9-fold and 22.8-fold increase respectively); whereas *Sp trm7Δ* [P_*nmt1*_ *trm7*^+^] strains grown under permissive conditions had relative mRNA levels very similar to WT cells. The GAAC induction levels in the *Sp trm7Δ* [P_*nmt1*_ *trm7*^+^] strains grown in repressing conditions were similar to those when WT *S*. *pombe* cells were treated with 10 mM or 30 mM 3-AT for 4 hours (Fig 7B, S7 Table). Thus, depletion of Trm7 in *S*. *pombe* resulted in little, if any, detectable defect in tRNA^Phe^ charging, but a robust induction of the GAAC pathway.

As in *S*. *cerevisiae*, *S*. *pombe* mutants lacking Cm_32_ or Gm_34_ of tRNA^Phe^ have GAAC responses that tracked with the growth defect. *Sp trm734Δ* strains grow relatively poorly [29], but not nearly as poorly as *trm7Δ* mutants, and had a partially activated GAAC response, with 6.5-fold and 6-fold increased relative mRNA levels of *lys4+* and *aor8+* (SPAC56E4.03) respectively, compared to 9.0-fold and 12.9-fold for *Sp trm7Δ* [P_*nmt1*_ *trm7*^+^] strains grown under repressive conditions. By contrast, *Sp trm732Δ* mutants have no obvious growth defect at 30°C—37°C [29], and had near wild type relative mRNA levels for *lys4+* and *aor8+* (SPAC56E4.03) (Fig 7C, S7 Table).

Further analysis showed that rescue of the growth defect of *S*. *pombe trm7* mutants reduced the GAAC response toward WT levels. As in *S*. *cerevisiae*, overproduction of *Sp* tRNA^Phe^ reduced the GAAC response as measured by relative mRNA levels of *lys4+* and *aor8+* (SPAC56E4.03) (Fig 7C, S7 Table), consistent with the rescue of the *Sp trm7Δ* growth defect we previously observed [29]. Furthermore, suppressors of the *Sp trm7Δ* growth defect behaved as in *S*. *cerevisiae*. We isolated *Sp trm7Δ* suppressors by plating *Sp trm7Δ* [P_*nmt1*_ *trm7*^+^] cells on media containing FOA, and each of two suppressors we analyzed had mutations in PheRS, and the suppressor strains in each case reduced the induction of the GAAC response (Fig 7C, S7 Table). These results suggest that lack of sufficient charged tRNA^Phe^ is also the main problem causing slow growth in *Sp trm7Δ* cells, despite the lack of detectable charging defect in acidic Northerns.

Based on the conserved induction of the GAAC response in *S*. *cerevisiae* and *S*. *pombe trm7Δ* mutants, we examined human lymphoblastoid cell lines with mutations in *FTSJ1* for an induced GAAC response by measuring mRNA levels of two Gcn2 dependent GAAC-regulated genes, *CTH* and *GADD153* [59]. Although WT control cell lines treated with the prolyl tRNA synthetase inhibitor halofuginone induced a significant GAAC response (S5A Fig) [60, 61], we obtained equivocal and inconclusive results for GAAC induction in the human lymphoblastoid *FTSJ1* cell lines, compared to the WT cell lines (S5B Fig).

## Discussion

Although standard acidic Northern analysis did not reveal significant reduced tRNA^Phe^ charging in *S*. *cerevisiae trm7Δ* mutants grown in rich media, we provided four lines of evidence supporting the conclusion that the growth defect of *trm7Δ* mutants is caused by reduced available charged tRNA^Phe^. First, contrary to our observations in rich media, in minimal media acidic Northern analysis revealed distinctly reduced tRNA^Phe^ charging in *trm7Δ* cells, charging was restored in each of three suppressors analyzed, and limiting phenylalanine exacerbated the *trm7Δ* growth defect. Thus, it seemed plausible that there was a subtler charging defect in rich media. Second, *trm7Δ* mutants activated a robust GAAC response in both rich media and minimal media, and activation of the GAAC response in rich media depended on Gcn2, which is known to sense uncharged tRNA [45, 62]. Third, each of 18 tested *trm7Δ* suppressors isolated in rich media suppressed the activation of the GAAC response found in *trm7Δ* mutants, and the vast majority had mutations that mapped to PheRS, arguing for the importance of increased charging for suppression of both the *trm7Δ* growth defect and the GAAC activation. Fourth, overproduction of PheRS also suppressed both the *trm7Δ* growth defect and GAAC activation, further implying that more charged tRNA^Phe^ could overcome the phenotypes of *trm7Δ* mutants. Ascribing the *trm7Δ* growth defect to reduced tRNA^Phe^ charging is also consistent with our previous observation that the steady state levels of tRNA^Phe^ were normal in *trm7Δ* mutants [28].

The effects of manipulation of EF-1A, *MAF1*, or tRNA^Phe^ gene dosage on suppression of *S*. *cerevisiae trm7Δ* phenotypes can also be interpreted in terms of tRNA^Phe^ charging or availability. The rescue of both the *trm7Δ* growth defect and GAAC activation by an extra copy of *TEF1* or *TEF2* could be due to the increased availability of the EF-1A:phe-tRNA^Phe^ complex for the translation machinery, achieved by increased overall binding of charged tRNA^Phe^ to EF-1A relative to PheRS, due to the tight binding constant of EF-1A for charged tRNA [63], or by preventing spontaneous deacylation of charged tRNA^Phe^ not bound by EF-1A, as demonstrated for EF-Tu [64]. The rescue of both the *trm7Δ* growth defect and GAAC activation by a *maf1* mutation is likely due to the observed increase in tRNA^Phe^ levels, consistent with the role of Maf1 as a negative regulator of pol III [49, 65], resulting in more charged tRNA^Phe^. Similarly, the rescue of both the *trm7Δ* growth defect [28] and GAAC induction by overexpression of tRNA^Phe^ is due to the 4.2-fold increase in tRNA^Phe^, and the commensurate increase in charged tRNA^Phe^. We note that there is also an increase in the ratio of charged:uncharged tRNA^Phe^ that occurs when tRNA^Phe^ is overexpressed or in a *maf1Δ* mutation; this likely results from the decreased relative usage of tRNA^Phe^ during translation when it is overproduced. We also note that the increase in uncharged tRNA^Phe^ that occurs when tRNA^Phe^ is overexpressed or in a *maf1Δ* mutation does not provoke the GAAC response. This result is consistent with the prevailing model that Gcn2 activation occurs in concert with the Gcn1-Gcn20 complex at the ribosome, triggered by entry of uncharged cognate tRNA at the A site independent of EF-1A [66–68]. Based on this model, the increased pools of charged tRNA would effectively outcompete the increased pool of uncharged tRNA for binding at the A-site when both are available, thus preventing activation of the GAAC response.

We have also shown that depletion of Trm7 in *S*. *pombe* resulted in a severe growth defect, and induced a robust GAAC response with no obvious alteration of tRNA^Phe^ charging as measured by acidic Northerns, and that suppressors of the growth defect reduced induction of the GAAC response and mapped to PheRS. Since the genes we assayed respond to the GAAC pathway when it is activated by uncharged tRNA, but not by other stimuli [58], we infer that *S*. *pombe trm7Δ* mutants, like *S*. *cerevisiae trm7Δ* mutants, behave as if they have uncharged tRNA.

It is intriguing that there was no discernible tRNA^Phe^ charging defect detected in acidic Northerns from *S*. *cerevisiae trm7Δ* mutants grown in rich media and in *S*. *pombe trm7Δ* [P_*nmt1*_ *trm7*^+^] mutants grown in repressing conditions, whereas mRNA levels analyzed from the same cultures showed robust induction of the GAAC response. There are at least three reasonable explanations of this observation. First, the tRNA^Phe^ charging defects may be too subtle to be detected by the acidic Northern assay, but can be effectively captured by the sensitive GAAC response. Acidic Northern analysis has been used extensively to measure charging since its initial description [36, 69]. However, quantification of uncharged tRNA might be particularly difficult for tRNA^Phe^ because of the higher background of uncharged tRNA^Phe^ in most RNA preps (Fig 1A; [70, 71]) and because of the possibility of incomplete yW modification of tRNA^Phe^ in WT cells grown in different conditions [72], which could interfere because of small mobility differences between uncharged tRNA^Phe^ with yW, and charged tRNA^Phe^ without yW. In this regard, it is not clear how much uncharged tRNA in the cell is required to activate the GAAC response for a given tRNA species [40]. Second, it is possible that tRNA^Phe^ is efficiently charged *in vivo*, but is sequestered from use in translation by some tRNA binding proteins, resulting in an increased probability that uncharged tRNA^Phe^ will bind at the ribosome A site and trigger the GAAC response. The tRNA^Phe^ might be sequestered in the nucleus by retrograde tRNA nuclear import [73, 74] or as an Msn5:EF-1A:phe-tRNA^Phe^ complex [75] somehow triggered by lack of the modifications, or perhaps sequestered in a stress granule [76]. However, it seems unlikely that charged tRNA^Phe^ is sequestered by binding as a product to PheRS, since overproduction of PheRS suppresses the growth defect and the GAAC response. Whether tRNA^Phe^ is subtly undercharged or is charged but effectively sequestered, the concordance of the *trm7Δ* growth defect and the robust GAAC response is striking in both *S*. *cerevisiae* and *S*. *pombe*, and suggests that they have the same root cause: lack of available charged tRNA^Phe^. A third explanation is that lack of Trm7 modifications causes ribosome stalling independent of uncharged tRNA, as reported in mouse mutants deficient in tRNA^Arg(UCU)^ and GTPBP2, a ribosome rescue factor [77].

The finding that a *gcn2Δ* or a *gcn4Δ* mutation partially improved growth of an *S*. *cerevisiae trm7Δ* strain indicates that some combination of the massively re-programmed expression pattern during the GAAC response [78] increases the stress on the *trm7Δ* mutants. This interpretation is consistent with models suggesting that constitutive activation of the GAAC response is deleterious to yeast [79, 80], as it may also be in metazoans based on the observation that inactivation of the GAAC response relieves TDP-43 toxicity in *Drosophila* and in mammalian neurons [81].

The GAAC activation we observed in *S*. *cerevisiae trm7Δ* mutants was more robust than each of the other anticodon loop modification mutants tested. The much more modest GAAC activation found in *kti12Δ* or *uba4Δ* mutants was very similar in magnitude to the Gcn2-independent GAAC activation found previously for disruption of the same mcm^5^s^2^U_34_ modification [82], and a similar modest GAAC activation was also detected in *mod5Δ* and *trm140Δ* mutants. These more modest GAAC activation levels are associated with mutants that have no obvious growth defect under these conditions; by contrast, the more substantial GAAC induction found in *pus3Δ* mutants is consistent with the known growth defect of *pus3Δ* mutants [18, 57]. Since a *pus3Δ* mutation impairs function of at least 3 of its 19 or more tRNA substrates in *S*. *cerevisiae* [18], it is possible that more than one tRNA is responsible for the GAAC induction. The extent of GAAC induction observed in these anticodon loop modification mutants is consistent with a recent study on transcriptome-wide analysis of roles for tRNA modifications by ribosome profiling [83].

It is unclear from our results why the *frs1* or *frs2* mutations that we identified from *S*. *cerevisiae trm7Δ* suppressors were all genetically dominant. Dominant PheRS mutations would be expected if *trm7Δ* mutants had a charging defect, since gain of function mutations are expected to be dominant. However, the *frs1* mutations map throughout the body of the protein, based on the human PheRS structure [84], bringing up the question of how scattered mutations all improve the function of the synthetase in *trm7Δ* mutants. As none of the *frs1* mutations localized to the editing domain, it is unlikely that these PheRS mutations reduce PheRS editing to inhibit GAAC induction, as observed for an *frs1* editing mutant grown under conditions of excess tyrosine relative to phenylananine [70, 71]).

Two models of PheRS function could explain the widespread locations of dominant *frs1* mutations among the *S*. *cerevisiae trm7Δ* suppressors. First, PheRS function could be reduced in *trm7Δ* mutants because of decreased recognition and binding of the hypomodified tRNA to PheRS, in which case the scattered *frs1* gain-of-function mutations would all act to improve interactions with tRNA. This model is plausible, and consistent with the principle of weak binding for efficient catalysis [85]. It is also formally possible in this model that the *frs1* mutations improve interaction between the two PheRS subunits, or that they improve stability or expression of the PheRS subunits, but these possibilities seem less likely to us because the mutations map all over the Frs1 subunit. Second, the charging activity of PheRS could be reduced in *trm7Δ* mutants because of increased binding of PheRS to hypomodified phe-tRNA^Phe^ and the consequent slow release of product, reducing the rate of multiple turnover reactions. Although in bacteria rate limiting product release is found in class I synthetases rather than class II synthetases like PheRS [86], this mechanism is in principle plausible for eukaryotic PheRS acting on tRNA^Phe^ lacking 2'-*O*-methylation. In this case, the scattered gain-of-function *frs1* mutations would all reduce interactions between PheRS and hypomodified tRNA^Phe^, promoting more effective release of charged tRNA^Phe^ from PheRS and increased overall charging. Both of these models call for specific interactions between the anticodon loop and PheRS, consistent with the known PheRS recognition of G_34_ [87], but the effects of Cm_32_ and Gm_34_ have not been tested [88, 89].

It is remarkable that in both *S*. *pombe* and *S*. *cerevisiae* the poor growth of *trm7Δ* mutants is associated with apparently complete tRNA^Phe^ charging but a robust GAAC response. Since these species diverged ∼330 to 420 million years ago [90], this result implies its generality among eukaryotes, to go along with the previously established conserved importance of tRNA^Phe^ as a Trm7 substrate in *S*. *pombe* and *S*. *cerevisiae*, the conserved anticodon loop modification circuitry of tRNA^Phe^ in *S*. *pombe*, *S*. *cerevisiae*, and humans, and the conserved favored importance of Gm_34_ in *S*. *pombe* and humans [28, 29, 34]. Moreover, all eukaryotic PheRS species appear to have similar recognition sets, since human and *S*. *cerevisiae* PheRS each recognize the same five residues [91], and tRNA^Phe^ from wheat germ or *S*. *pombe* is charged by *S*. *cerevisiae* PheRS nearly as effectively as the native substrates [92]. Although our preliminary analysis of the GAAC response in human lymphoblastoid cell lines with mutations in *FTSJ1* yielded equivocal results, this analysis might require specialized cell types to explain the non-syndromic nature of NSXLID [30–34], or the cell lines may have accumulated secondary lesions that mask the GAAC induction. Based on this high degree of conservation of the biology of Trm7 and PheRS, we speculate that GAAC activation will be widely conserved in *trm7* mutants in eukaryotes, including metazoans, and might play a role in NSXLID due to lesions in human *FTSJ1*.

## Materials and methods

### Yeast strains

Yeast strains used in this study are listed in S1 and S8 Tables. *trm7Δ* supp 1 to 10 were isolated from yMG105 (MATa, *trm7Δ*::*ble*^*R*^) strain, and supp 11 to 21 from yMG107 (MATα, *trm7Δ*::*ble*^*R*^) strain. For all other experiments, *trm7Δ* mutants were freshly derived from yMG348-1 *trm7Δ*::*ble*^*R*^ [*CEN URA3 TRM7*] each time before use, by growing yMG348-1 in YPD media overnight followed by streaking on media containing FOA to select against the *URA3* plasmid. The WT His^+^ strains were derived from BY4741 by PCR amplification of *HIS3* with its 5' and 3' flanking sequence, followed by linear transformation, selection on SD-His and PCR verification. *PHA2*, *GCN2*, *KTI12*, *UBA4*, *MAF1*, and *TEF2* were deleted by PCR amplification of DNA from the appropriate YKO collection *kanMX* strains using oligomers containing sequences 5' and 3' of the gene [93], followed by linear transformation and selection on YPD media containing 300 mg/L geneticin. *GCN4* was deleted by PCR amplification of the *hyg*^*R*^ marker, followed by linear transformation and selection on YPD media containing 300 mg/L hygromycin B. All *trm7Δ* double-mutant strains were constructed similarly by PCR amplification, linear transformation into yMG348-1 *trm7Δ*::*ble*^*R*^ [*CEN URA3 TRM7*], and selection against the *URA3* plasmid by streaking on media containing FOA.

The haploid *S*. *pombe trm7Δ*::*kanMX* [*ura4*^*+*^ P_*nmt1*_ *trm7*^*+*^] (yMG1052A) strain was generated as previously described [29] and used for isolation of suppressors. The haploid *trm7Δ*::*kanMX* [*LEU2* P_*nmt1* low strength_ *trm7*^*+*^] (yMG1541) strain was generated by transformation of yMG1052A with a *LEU2* P_*nmt1* low strength_ *sp trm7*^*+*^ plasmid (pMG527B), and selection against the *ura4*^*+*^ plasmid by streaking on media containing FOA, and was used for experiments in which Trm7 was depleted with thiamine.

For all experiments in which two or more strains with the same genotype are analyzed, these samples are biological replicates.

### Plasmids

Plasmids used in this study are listed in S9 Table. Plasmids for *FRS1* and/or *FRS2* expression were derived from pBG2619, which is a [2μ P_*GAL1*,*10*_ LIC] dual ORF expression plasmid. In this plasmid, expression of one ORF is under P_*GAL1*_ control with a C-terminal PT tag, containing 3C site-HA epitope-His_6_-ZZ domain of protein A, and expression of the second ORF is under P_*GAL10*_ control with no tag [94]. *CEN* plasmids were constructed by ligation-independent clone (LIC) of genes containing their own 5' and 3' flanking sequence into pAVA581 (*LEU2*) or pAVA579 (*URA3*) [94].

### Northern blot analysis

*S*. *cerevisiae* strains were grown at 30°C to mid-log phase in either rich media or minimal media as indicated. *S*. *pombe* strains were grown at 30°C to mid-log phase in EMM supplemented with 225 mg/L adenine, lysine, histidine, leucine, and uracil. To analyze WT and *trm7Δ* [P_*nmt1*_ *trm7*^*+*^] strains under repressive growth conditions, thiamine was supplemented to the media at 5 mg/L. For either *S*. *cerevisiae* or *S*. *pombe*, bulk RNA was prepared from ~4 OD pellets using glass beads, and RNA was resolved on acrylamide gels and analyzed by hybridization as previously described [3]. For analysis of charging, RNA was prepared and resolved under acidic conditions as described [3].

### Real-time quantitative PCR

Strains were grown in triplicate to mid-log phase as described above for Northern blot analysis. Bulk RNA was prepared from 5–10 OD pellets using glass beads, treated with DNase, reverse transcribed, and the resulting cDNA was amplified and analyzed as previously described [95].

### Whole genome sequencing

Whole genome sequencing was done at the UR Genomics Research Center at a read depth of greater than 100-reads.

## Supporting information

S1 Fig Overproduction of tRNA^Phe^ fully rescues *trm7Δ* growth defect on both rich and minimal media. WT or *trm7Δ* strains containing a high-copy *LEU2* plasmid expressing *TRM7*, tRNA^Phe^, tRNA^Trp^, tRNA^Leu(UAA)^, or a vector as indicated were grown in SD-Leu, analyzed by spotting to plates as indicated, and incubated for 2 d at 30°C.
(TIF)

S2 Fig Overproduction of PheRS restores tRNA^Phe^ charging levels in *trm7Δ* mutants to WT levels. WT and *trm7Δ* strains containing a high-copy [2μ *LEU2*] plasmid expressing *FRS1*and *FRS2* under control of the P_*GAL*_ promoter, or a vector control, were grown in S-Leu medium containing raffinose and galactose, and then RNA was isolated under acidic conditions and analyzed for charging as in Fig 1(*A*).
(TIF)

S3 Fig An additional copy of EF-1A does not alter tRNA^Phe^ charging in *trm7Δ* mutants. WT or *trm7Δ* strains containing a [*CEN LEU2*] plasmid expressing *TEF1* or *TEF2*, or a vector control, were grown in SD-Leu, and then RNA was isolated under acidic conditions and analyzed for charging as in Fig 1(*A*).
(TIF)

S4 Fig Overproduction of tRNA^Phe^ or a *maf1* deletion results in increased levels of tRNA^Phe^ and tRNA^Phe^ charging in *trm7Δ* mutants. **(A)** WT, *trm7Δ*, or *tyw1Δ* strains containing a high-copy [2μ *LEU2*] plasmid expressing *tF(GAA)*, or a vector control, were grown in SD-Leu, and then RNA was isolated under acidic conditions and analyzed for charging as in Fig 1(*A*). a, b, 1.5 μg RNA analyzed; b/4, 0.375 μg analyzed. Relative expression of *tF(GAA)* represents *tF(GAA)* expression normalized to that of *tG(GCC)*, and then normalized to expression in WT [vec], itself normalized to *tG(GCC)*. WT, *trm7Δ*, and *tyw1Δ* strains overexpressing *tF(GAA)* have 3.7-, 4.2-, and 2.6-fold more tRNA^Phe^ respectively than the corresponding vector control strains. **(B)** Strains as indicated were grown in minimal (SD complete) media to log phase or stationary phase, and then RNA was isolated under acidic conditions and analyzed for charging as in Fig 1(*A*).
(TIF)

S5 Fig GAAC induction in human lymphoblastoid *FTSJ1* cell lines is inconclusive. **(A)** A WT control cell line treated with halofuginone induces a significant GAAC response. A WT control cell line was grown as previously described [34] and then treated with halofuginone (HF) at indicated concentrations for 4 hours. Bulk RNA was then extracted, and analyzed by RT-qPCR for mRNA levels of Gcn2-dependent GAAC-regulated genes, *CTH* and *GADD153*, normalized to those of nonregulated *GAPDH*. **(B)** GAAC induction in human lymphoblastoid *FTSJ1* cell lines. WT control cell lines and *FTSJ1* cell lines as indicated [34] were examined for GAAC induction as in A.
(TIF)

S1 Table *frs1* and *frs2* mutations identified in *trm7Δ* suppressors. (PDF)

S2 Table Relative mRNA levels in Fig 2. (PDF)

S3 Table Relative mRNA levels in Fig 3. (PDF)

S4 Table Relative mRNA levels in Fig 4B. (PDF)

S5 Table Relative mRNA levels in Fig 5A. (PDF)

S6 Table Relative mRNA levels in Fig 6. (PDF)

S7 Table Relative mRNA levels in Fig 7. (PDF)

S8 Table Strains used in this study. (PDF)

S9 Table Plasmids used in this study. (PDF)
