# Supplementary figures and images for: Lack of 2'-O-methylation in the tRNA anticodon loop of two phylogenetically distant yeast species activates the general amino acid control pathway
Source: PLoS Genet. 2018 Mar 29;14(3):e1007288. doi: 10.1371/journal.pgen.1007288 (PMC5892943; doi:10.1371/journal.pgen.1007288)

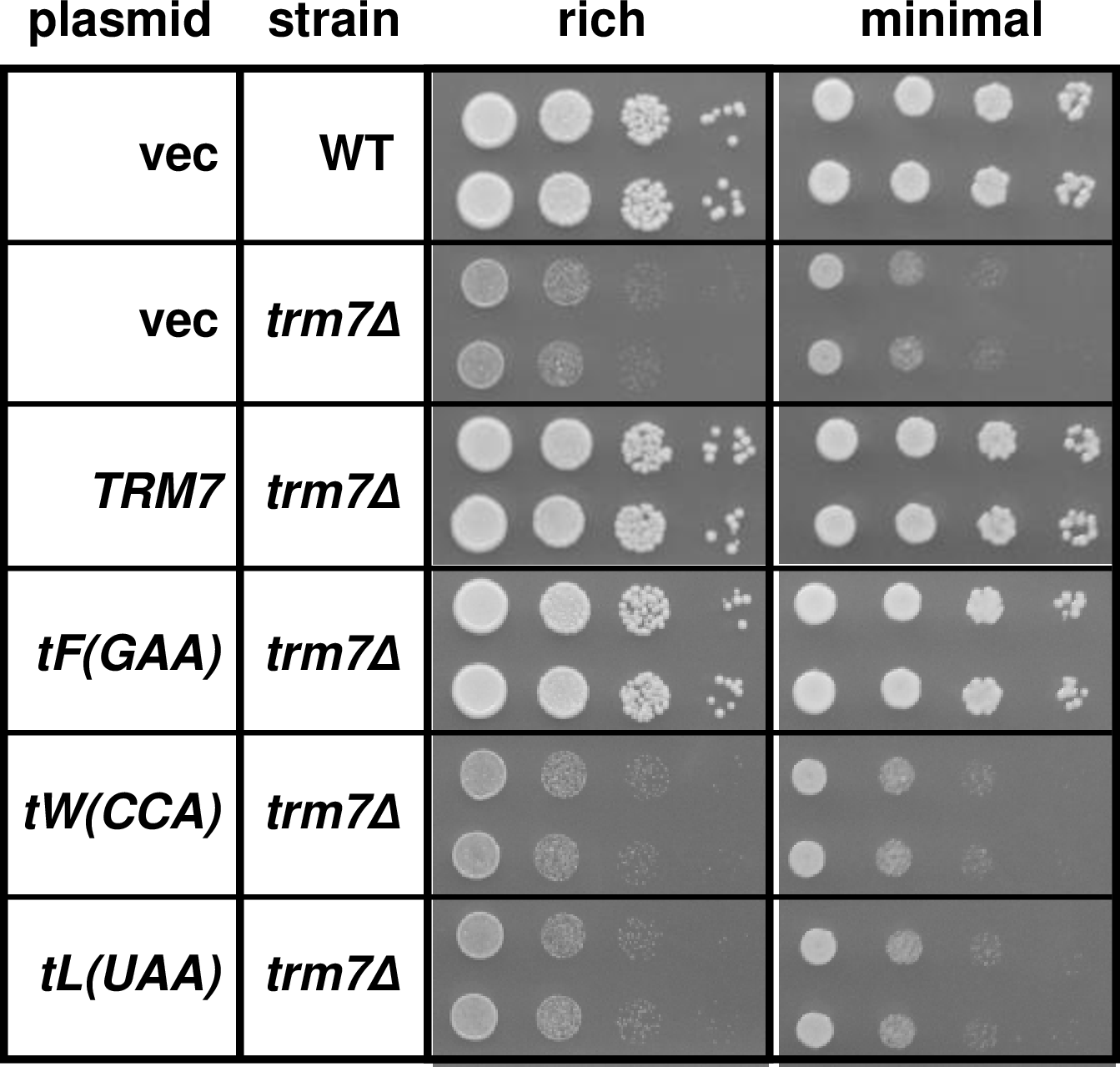

Supplement: S1 Fig — WT or trm7Δ strains containing a high-copy LEU2 plasmid expressing TRM7, tRNAPhe, tRNATrp, tRNALeu(UAA), or a vector as indicated were grown in SD-Leu, analyzed by spotting to plates as indicated, and incubated for 2 d at 30°C. (TIF) [file pgen.1007288.s001.tif]

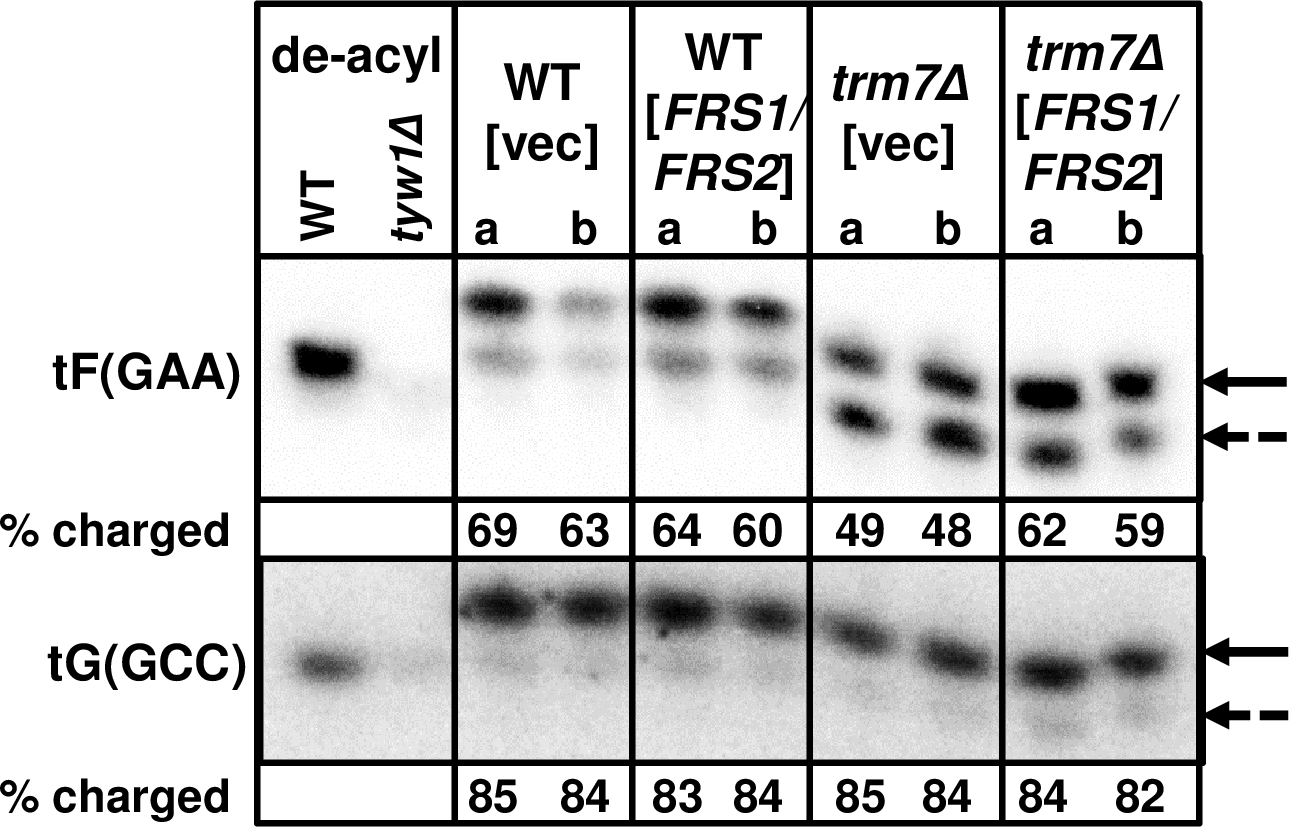

Supplement: S2 Fig — WT and trm7Δ strains containing a high-copy [2μ LEU2] plasmid expressing FRS1and FRS2 under control of the PGAL promoter, or a vector control, were grown in S-Leu medium containing raffinose and galactose, and then RNA was isolated under acidic conditions and analyzed for charging as in Fig 1(A). (TIF) [file pgen.1007288.s002.tif]

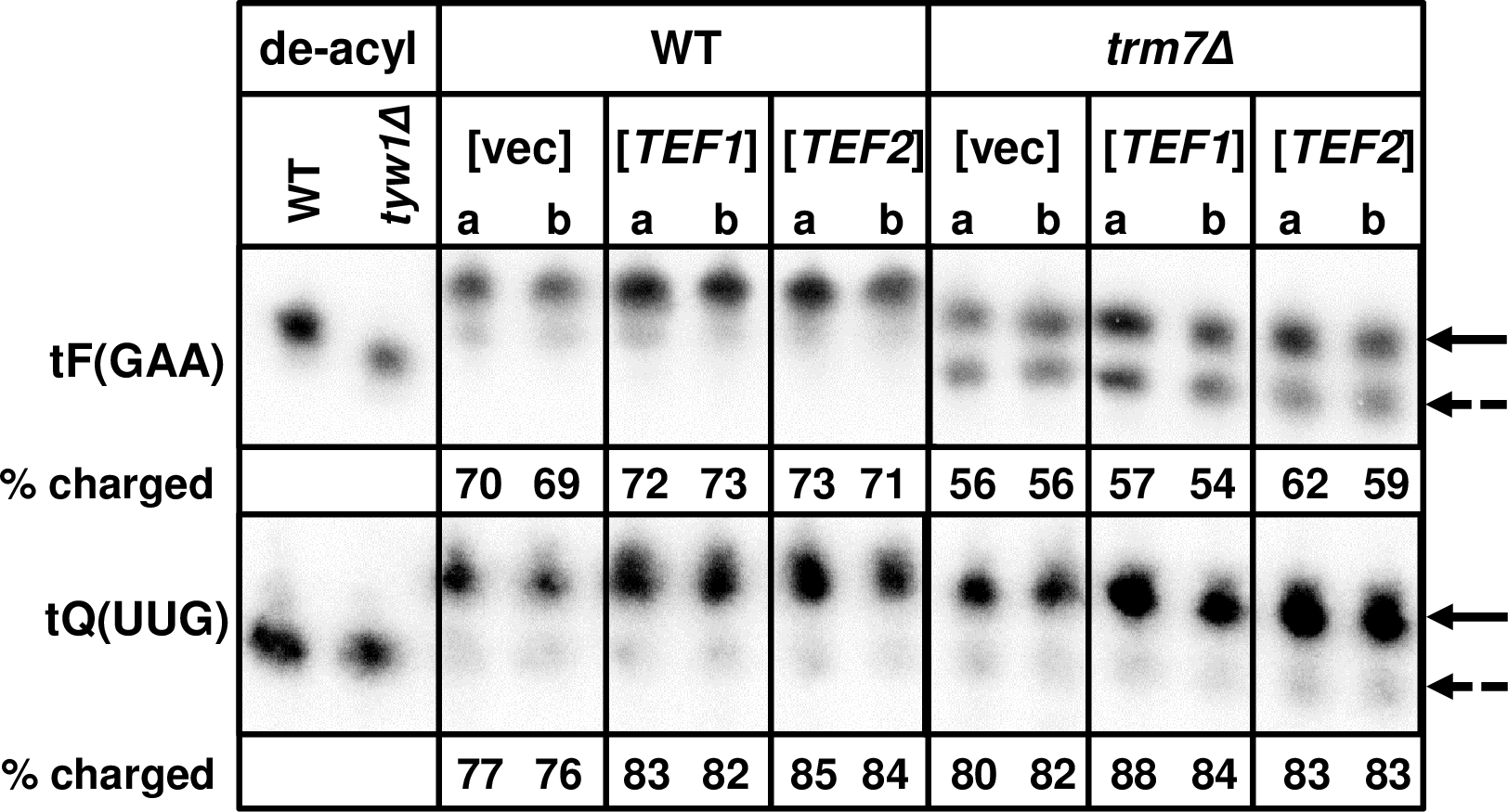

Supplement: S3 Fig — WT or trm7Δ strains containing a [CEN LEU2] plasmid expressing TEF1 or TEF2, or a vector control, were grown in SD-Leu, and then RNA was isolated under acidic conditions and analyzed for charging as in Fig 1(A). (TIF) [file pgen.1007288.s003.tif]

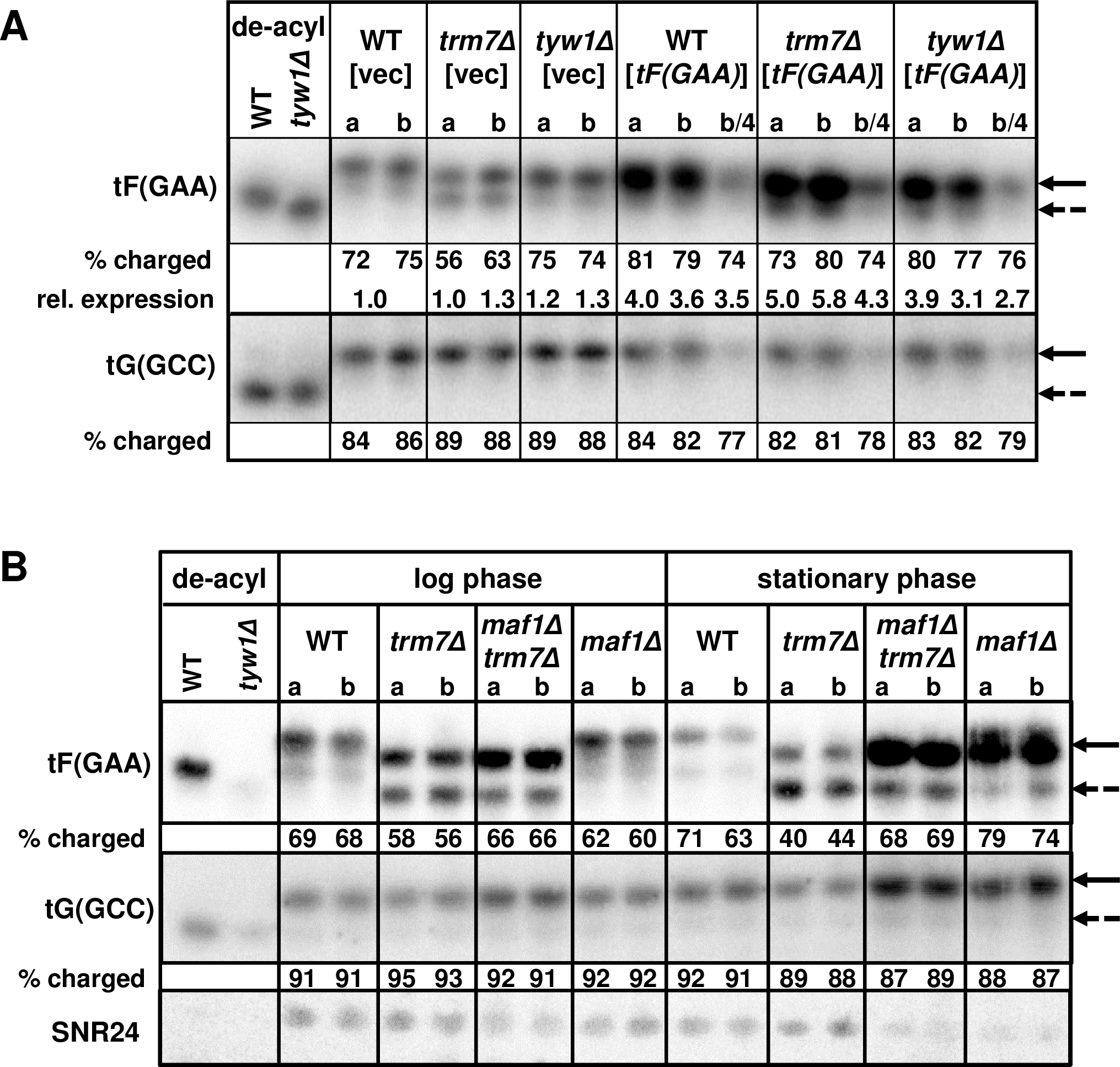

Supplement: S4 Fig — (A) WT, trm7Δ, or tyw1Δ strains containing a high-copy [2μ LEU2] plasmid expressing tF(GAA), or a vector control, were grown in SD-Leu, and then RNA was isolated under acidic conditions and analyzed for charging as in Fig 1(A). a, b, 1.5 μg RNA analyzed; b/4, 0.375 μg analyzed. Relative expression of tF(GAA) represents tF(GAA) expression normalized to that of tG(GCC), and then normalized to expression in WT [vec], itself normalized to tG(GCC). WT, trm7Δ, and tyw1Δ strains overexpressing tF(GAA) have 3.7-, 4.2-, and 2.6-fold more tRNAPhe respectively than the corresponding vector control strains. (B) Strains as indicated were grown in minimal (SD complete) media to log phase or stationary phase, and then RNA was isolated under acidic conditions and analyzed for charging as in Fig 1(A). (TIF) [file pgen.1007288.s004.tif]

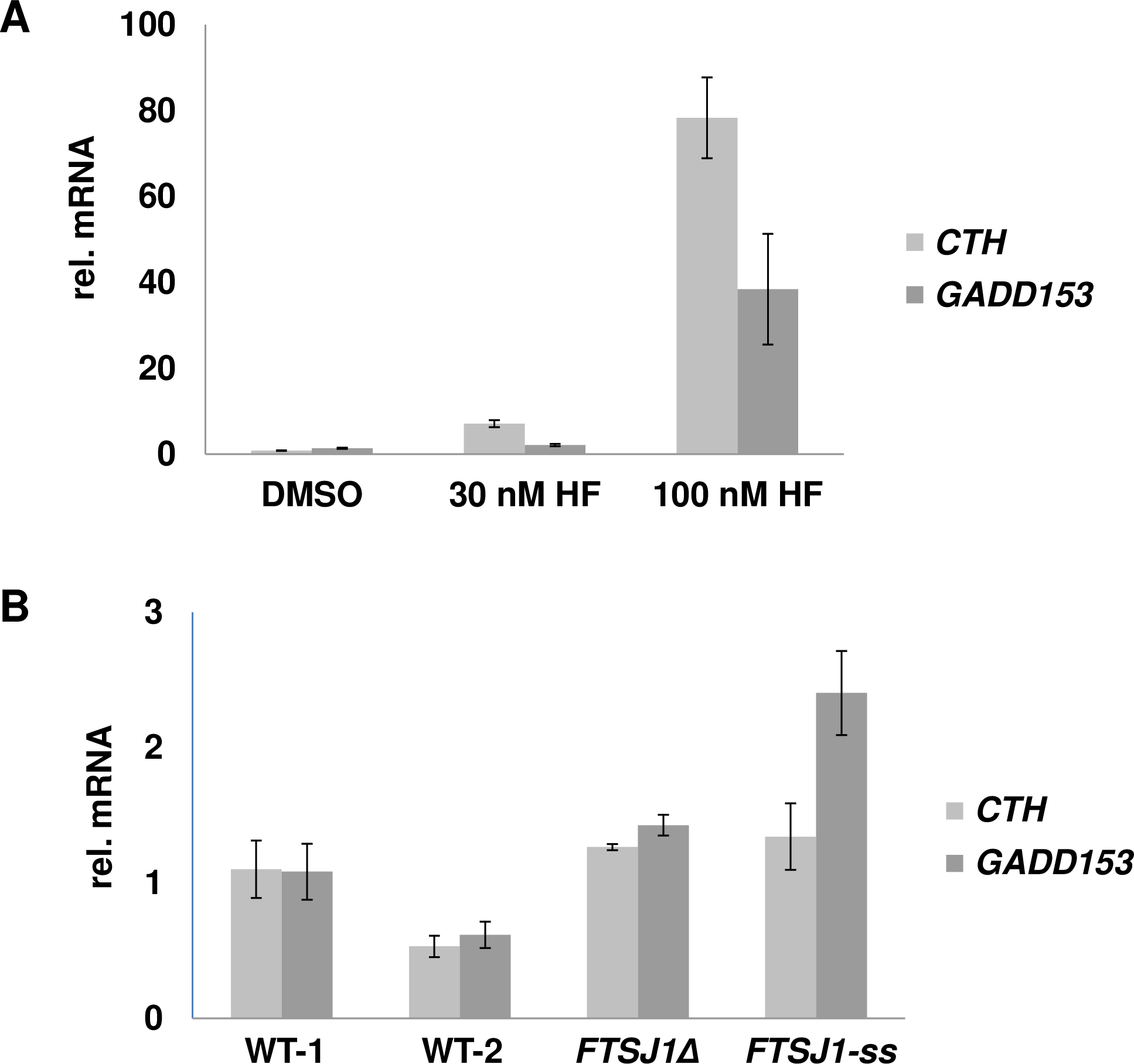

Supplement: S5 Fig — (A) A WT control cell line treated with halofuginone induces a significant GAAC response. A WT control cell line was grown as previously described [34] and then treated with halofuginone (HF) at indicated concentrations for 4 hours. Bulk RNA was then extracted, and analyzed by RT-qPCR for mRNA levels of Gcn2-dependent GAAC-regulated genes, CTH and GADD153, normalized to those of nonregulated GAPDH. (B) GAAC induction in human lymphoblastoid FTSJ1 cell lines. WT control cell lines and FTSJ1 cell lines as indicated [34] were examined for GAAC induction as in A. (TIF) [file pgen.1007288.s005.tif]
