# Supplementary material for: Lack of 2'-O-methylation in the tRNA anticodon loop of two phylogenetically distant yeast species activates the general amino acid control pathway
Source: PLoS Genet. 2018 Mar 29;14(3):e1007288. doi: 10.1371/journal.pgen.1007288 (PMC5892943; doi:10.1371/journal.pgen.1007288)
Supplement: S1 Table — (PDF) [file pgen.1007288.s006.pdf]

Table S1. ***frs1*** **and** ***frs2*** **mutations identified in** ***trm7Δ*** **suppressors.**

Strain	*frs1*	*frs2*
trm7Δ supp 1	E415K	
trm7Δ supp 2	Y532C	
trm7Δ supp 3	A549T	
trm7Δ supp 4	V361A	
trm7Δ supp 5		V101F
trm7Δ supp 6	WT	WT
trm7Δ supp 7	L567F	
trm7Δ supp 8		D275G
trm7Δ supp 9	D55H	
trm7Δ supp 10	N486S	
trm7Δ supp 11	A58S	
trm7Δ supp 12	WT	WT
trm7Δ supp 13	A549T	
trm7Δ supp 14		L265V
trm7Δ supp 15	V430A	
trm7Δ supp 16		L265I
trm7Δ supp 17	D579T	
trm7Δ supp 18		V267I
trm7Δ supp 19	G500A	
trm7Δ supp 20		P268T
trm7Δ supp 21	D62N	
