# Supplementary material for: Lack of 2'-O-methylation in the tRNA anticodon loop of two phylogenetically distant yeast species activates the general amino acid control pathway
Source: PLoS Genet. 2018 Mar 29;14(3):e1007288. doi: 10.1371/journal.pgen.1007288 (PMC5892943; doi:10.1371/journal.pgen.1007288)
Supplement: S2 Table — (PDF) [file pgen.1007288.s007.pdf]

Table S2. Relative mRNA levels in Fig. 2.

Fig. 2	media	strain	*HIS5*/*ACT1*	*LYS1*/*ACT1*
A	rich	WT	1.0 ± 0.3	1.0 ± 0.3
		trm7Δ [*TRM7*]	1.7 ± 0.6	1.9 ± 0.5
		trm7Δ	27.8 ± 3.0	90.9 ± 13.4
	synthetic minimal	WT	1.0 ± 0.1	2.9 ± 0.3
		trm7Δ [*TRM7*]	3.1 ± 0.2	7.9 ± 1.1
		trm7Δ	17.1 ± 1.6	43.2 ± 2.1
B	rich	WT	1.0 ± 0.3	
		trm7Δ	50.5 ± 10.5	
	0 mM 3-AT	WT His$^+$	1.0 ± 0.1	
	10 mM 3-AT		44.3 ± 8.1	
	100 mM 3-AT		62.1 ± 0.2	
C	rich	WT	1.0 ± 0.4	
		trm7Δ	88.0 ± 9.6	
		gcn2Δ	1.3 ± 0.1	
		trm7Δ gcn2Δ	1.7 ± 0.1	
		gcn4Δ	0.8 ± 0.2	
		trm7Δ gcn4Δ	1.9 ± 0.2	
E	rich	WT	1.0 ± 0.1	
		trm7Δ	46.8 ± 2.3	
		trm732Δ	0.9 ± 0.2	
		trm734Δ	1.0 ± 0.1	
		tyw1Δ	0.8 ± 0.2	
		trm732Δ tyw1Δ	1.5 ± 0.2	
		trm734Δ tyw1Δ	1.2 ± 0.3	
		trm732Δ trm734Δ	43.2 ± 5.1	
