# Supplementary material for: Lack of 2'-O-methylation in the tRNA anticodon loop of two phylogenetically distant yeast species activates the general amino acid control pathway
Source: PLoS Genet. 2018 Mar 29;14(3):e1007288. doi: 10.1371/journal.pgen.1007288 (PMC5892943; doi:10.1371/journal.pgen.1007288)
Supplement: S3 Table — (PDF) [file pgen.1007288.s008.pdf]

Table S3. Relative mRNA levels in Fig. 3.

Fig. 3	strain	*HIS5/ACT1*
A	WT	1.0 ± 0.2
	trm7Δ	37.3 ± 5.7
	frs1-E415K	1.3 ± 0.1
	frs1-Y532C	1.6 ± 0.5
	frs1-A549T	1.8 ± 0.6
	frs1-V361A	2.4 ± 0.3
	frs2-V101F	1.4 ± 0.2
	supp 6	4.7 ± 0.6
	frs2-D275G	1.3 ± 0.1
	frs1-D55H	1.3 ± 0.1
	frs1-N486S	1.6 ± 0.1
	WT	1.0 ± 0.2
	trm7Δ	36.1 ± 1.6
	frs1-A58S	1.8 ± 0.9
	supp 12	5.5 ± 1.4
	frs2-L265V	1.7 ± 0.2
	frs1-V430A	3.7 ± 0.2
	frs1-D579T	2.0 ± 0.2
	frs2-V267I	1.1 ± 0.2
	frs1-G500A	1.6 ± 0.4
	frs2-P268T	2.1 ± 0.5
	frs1-D62N	2.1 ± 0.1
C	WT [vec]	1.0 ± 0.5
	trm7Δ [vec]	17.8 ± 1.9
	trm7Δ [*FRS1/FRS2*]	4.9 ± 0.4
	trm7Δ [*FRS1*]	23.2 ± 4.6
	trm7Δ [*FRS2*]	19.4 ± 2.7
