# Supplementary material for: Lack of 2'-O-methylation in the tRNA anticodon loop of two phylogenetically distant yeast species activates the general amino acid control pathway
Source: PLoS Genet. 2018 Mar 29;14(3):e1007288. doi: 10.1371/journal.pgen.1007288 (PMC5892943; doi:10.1371/journal.pgen.1007288)
Supplement: S4 Table — (PDF) [file pgen.1007288.s009.pdf]

Table S4. Relative mRNA levels in Fig. 4B.

strain	*HIS5*/*ACT1*
WT [vec]	1.0 ± 0.2
WT [*TEF1*]	0.6 ± 0.1
WT [*TEF2*]	1.2 ± 0.5
trm7Δ [vec]	3.7 ± 0.5
trm7Δ [*TEF1*]	2.2 ± 0.4
trm7Δ [*TEF2*]	2.8 ± 0.4
