# Supplementary material for: Lack of 2'-O-methylation in the tRNA anticodon loop of two phylogenetically distant yeast species activates the general amino acid control pathway
Source: PLoS Genet. 2018 Mar 29;14(3):e1007288. doi: 10.1371/journal.pgen.1007288 (PMC5892943; doi:10.1371/journal.pgen.1007288)
Supplement: S5 Table — (PDF) [file pgen.1007288.s010.pdf]

Table S5. Relative mRNA levels in Fig. 5A.

strain	*HIS5*/*ACT1*
WT [vec]	1.0 ± 0.2
WT [*tF(GAA)*]	1.2 ± 0.3
WT [*tL(UAA)*]	1.1 ± 0.1
WT [*tW(CCA)*]	1.1 ± 0.5
trm7Δ [vec]	3.7 ± 0.5
trm7Δ [*tF(GAA)*]	1.0 ± 0.4
trm7Δ [*tL(UAA)*]	4.6 ± 0.5
trm7Δ [*tW(CCA)*]	5.0 ± 0.6
