# Supplementary material for: Lack of 2'-O-methylation in the tRNA anticodon loop of two phylogenetically distant yeast species activates the general amino acid control pathway
Source: PLoS Genet. 2018 Mar 29;14(3):e1007288. doi: 10.1371/journal.pgen.1007288 (PMC5892943; doi:10.1371/journal.pgen.1007288)
Supplement: S6 Table — (PDF) [file pgen.1007288.s011.pdf]

Table S6. Relative mRNA levels in Fig. 6.

strain	*HIS5*/*ACT1*
WT	1.0 ± 0.4
trm7Δ	116.0 ± 3.2
mod5Δ	2.2 ± 0.2
trm140Δ	1.8 ± 0.9
kti12Δ	3.2 ± 0.9
uba4Δ	2.3 ± 0.1
pus3Δ	15.7 ± 1.7
