# Supplementary material for: Lack of 2'-O-methylation in the tRNA anticodon loop of two phylogenetically distant yeast species activates the general amino acid control pathway
Source: PLoS Genet. 2018 Mar 29;14(3):e1007288. doi: 10.1371/journal.pgen.1007288 (PMC5892943; doi:10.1371/journal.pgen.1007288)
Supplement: S7 Table — (PDF) [file pgen.1007288.s012.pdf]

Table S7. Relative mRNA levels in Fig. 7.

Fig. 7	strain	media	*lys4*$^{+}$/*act1*$^{+}$	*aro8*$^{+}$ *(SPBC1773.13)* /*act1*$^{+}$	*aro8*$^{+}$ *(SPAC56E4.03)* /*act1*$^{+}$
B	WT	- thiamine	1.0 ± 0.5	1.0 ± 0.8	1.0 ± 0.5
		+ thiamine	1.6 ± 1.0	0.6 ± 0.3	2.1 ± 1.1
	trm7Δ [P_{nmt1} *trm7*$^{+}$]	- thiamine	2.0 ± 0.5	1.4 ± 0.3	2.8 ± 0.9
		+ thiamine	14.7 ± 1.2	6.9 ± 1.5	22.8 ± 3.5
	WT	0 mM 3-AT	1.0 ± 0.5	1.0 ± 0.8	1.0 ± 0.5
		10 mM 3-AT	18.5 ± 5.7	11.8 ± 6.0	27.2 ± 6.5
		30 mM 3-AT	24.2 ± 4.9	15.6 ± 2.0	29.0 ± 1.7
C	WT	- thiamine	1.0 ± 0.2		1.0 ± 0.3
	trm732Δ	- thiamine	1.7 ± 0.5		1.2 ± 0.1
	trm734Δ	- thiamine	6.0 ± 0.9		6.5 ± 1.2
	trm7Δ [P_{nmt1} *trm7*$^{+}$]	- thiamine	3.2 ± 0.8		2.5 ± 0.1
		+ thiamine	12.9 ± 2.5		9.0 ± 1.0
	trm7Δ [*tF(GAA)*]	- thiamine	3.3 ± 0.8		3.5 ± 0.5
	trm7Δ frs2-F211L	- thiamine	3.3 ± 0.1		3.3 ± 0.1
	trm7Δ frs1-I406L	- thiamine	1.5 ± 0.5		0.7 ± 0.2
