# Supplementary material for: Lack of 2'-O-methylation in the tRNA anticodon loop of two phylogenetically distant yeast species activates the general amino acid control pathway
Source: PLoS Genet. 2018 Mar 29;14(3):e1007288. doi: 10.1371/journal.pgen.1007288 (PMC5892943; doi:10.1371/journal.pgen.1007288)
Supplement: S8 Table — (PDF) [file pgen.1007288.s013.pdf]

Table S8. Strains used in this study.

Strain name	Parent strain	Genotype	Source
<u>*S. cerevisiae*</u>			
BY4741		*MATa his3Δ1 leu2Δ0 met15Δ0 ura3Δ0*	
BY4742		*MATα his3Δ1 leu2Δ0 lys2Δ0 ura3Δ0*	
yMG105	BY4741	*trm7Δ::ble*^{R}	(Guy et al. 2012)
yMG107	BY4742	*trm7Δ::ble*^{R}	This study
YJYW2566	BY4741	*MATa leu2Δ0 met15Δ0 ura3Δ0*	This study
yMG348-1	BY4741	*trm7Δ::ble*^{R} [*CEN URA3 TRM7*]	(Guy et al. 2012)
yMG814-1	BY4741	*trm732Δ::ble*^{R}	(Guy et al. 2012)
yMG724-5	BY4741	*trm734Δ::ble*^{R}	(Guy et al. 2012)
yMAB109	BY4741	*tyw1Δ ::kanMX*	(Guy et al. 2012)
yMG818-1	yMG724-5	*trm734Δ::ble*^{R} *trm732Δ::kanMX* [*CEN URA3 TRM734*]	(Guy et al. 2012)
yMG956-1	yMG724-5	*trm734Δ::ble*^{R} *tyw1Δ::kanMX* [*CEN URA3 TRM734*]	(Guy et al. 2012)
yMG899-1	yMG814-1	*trm732Δ::ble*^{R} *tyw1Δ::kanMX* [*CEN URA3 TRM732*]	(Guy et al. 2012)
YK1277	BY4741	*pha2Δ ::kanMX*	This study
YK1282	yMG348-1	*trm7Δ::ble*^{R} *pha2Δ ::kanMX* [*CEN URA3 TRM7*]	This study
YLH1421	BY4741	*gcn2Δ::kanMX*	This study
YLH1422	yMG348-1	*trm7Δ::ble*^{R} *gcn2Δ::kanMX* [*CEN URA3 TRM7*]	This study
YK1281	BY4741	*gcn4Δ::hygro*^{R}	This study
YK1279	yMG348-1	*trm7Δ::ble*^{R} *gcn4Δ::hygro*^{R} [*CEN URA3 TRM7*]	This study
ySD179	BY4742	*trm140Δ::ble*^{R}	(Han et al. 2017)
ySD844	BY4741	*mod5Δ::kanMX*	(Han et al. 2017)
YK428-1	BY4741	*pus3Δ::ble*^{R}	(Han et al. 2015)
YLH442	BY4741	*kti12Δ::kanMX*	This study
YLH443	BY4741	*uba4Δ::kanMX*	This study
YK1424	BY4741	*maf1Δ::kanMX*	This study
yMG323-1	BY4741	*tef2Δ::kanMX*	This study
yMG342-2	yMG348-1	*trm7Δ::ble*^{R} *tef2Δ::hygro*^{R} [*CEN URA3 TRM7*]	This study
YK1423	yMG348-1	*trm7Δ::ble*^{R} *maf1Δ::kanMX* [*CEN URA3 TRM7*]	This study
<u>*S. pombe*</u>			
SP286		WT diploid *ade6*-M210/*ade6*-M216 *ura4*-D18/*ura4*-D18 *leu1-32*/*leu1-32*	Bioneer
yMG957B	SP286	WT haploid	(Guy and Phizicky 2015)
yMG1052A	SP286	*trm7Δ::kanMX* [*ura4*$^+$ P$_{nmt1}$ *trm7*$^+$]	(Guy and Phizicky 2015)
yMG1541	SP286	*trm7Δ::kanMX* [*LEU2* P$_{nmt1\ \mathrm{low\ strength}}$ *trm7*$^+$]	This study
yMG1289-1	SP286	*trm734Δ::kanMX* [*ura4*$^+$ P$_{trm734}$ *trm734*$^+$]	(Guy and Phizicky 2015)
yMG958B	SP286	*trm732Δ::kanMX*	(Guy and Phizicky 2015)
