# Supplementary material for: Lack of 2'-O-methylation in the tRNA anticodon loop of two phylogenetically distant yeast species activates the general amino acid control pathway
Source: PLoS Genet. 2018 Mar 29;14(3):e1007288. doi: 10.1371/journal.pgen.1007288 (PMC5892943; doi:10.1371/journal.pgen.1007288)
Supplement: S9 Table — (PDF) [file pgen.1007288.s014.pdf]

Table S9. Plasmids used in this study.

Plasmid	Parent	Description	Source
pAVA579		*CEN URA3* LIC	(Quartley et al. 2009)
pMG13	pAVA579	*CEN URA3 TRM7*	(Guy et al. 2012)
pBP2A	pAVA579	*CEN URA3 TRM734*	(Guy et al. 2012)
pMG236A	pAVA579	*CEN URA3 TRM732*	This study
pBG2619		2µ *LEU2* $P_{GAL1,10}$ LIC	(Quartley et al. 2009)
pMG237E	pBG2619	2µ *LEU2* P_{GAL} *FRS1-PT FRS2-NT*	This study
pMG189A	pBG2619	2µ *LEU2* P_{gal} *FRS1-NT*	This study
pMG232A	pBG2619	2µ *LEU2* P_{gal} *FRS2-NT*	This study
pMAB813A		2µ *LEU2 tH(GUG)*	(Guy et al. 2012)
pMG16A	pMAB813A	2µ *LEU2 tF(GAA)*	(Guy et al. 2012)
pMG18B	pMAB813A	2µ *LEU2 tW(CCA)*	(Guy et al. 2012)
pMG24A	pMAB813A	2µ *LEU2 tL(UAA)*	(Guy et al. 2012)
AVA 581		*CEN LEU2* LIC	(Quartley et al. 2009)
EJD066-1	AVA 581	*CEN LEU2 TEF1*	This study
EJD067-1	AVA 581	*CEN LEU2 TEF2*	This study
pREP4X		*ura4*$^{+}$ P_{nmt1}	(Forsburg 1993)
pMG360A	pREP4X	*ura4*$^{+}$ P_{nmt1} *sp trm7*$^{+}$	(Guy and Phizicky 2015)
pMG426G	pREP4X	*ura4*$^{+}$ P_{trm734} *sp trm734*$^{+}$	(Guy and Phizicky 2015)
pREP81X		*LEU2* $P_{nmt1\ \text{low strength}}$	(Forsburg 1993)
pMG527B	pREP81X	*LEU2* $P_{nmt1\ \text{low strength}}$ *sp trm7*$^{+}$	(Guy and Phizicky 2015)
pREP3X		*LEU2* P_{nmt1}	(Forsburg 1993)
pMG308C	pREP3X	*LEU2 sp tF(GAA)*	(Guy and Phizicky 2015)
